# Beyond redlining: Gentrification, displacement, disadvantages, and exclusivity predict urban environmental and health inequities

**DOI:** 10.1073/pnas.2422760122

**Published:** 2026-09-14

**Authors:** Idowu Ajibade, Kevan B. Moffett, Jason Maxfield, Kate Gregory, Axcelle Bell, Jackson Voelkel, Todd Rosenstiel, Aaron R. Ramirez

**Affiliations:** ^a^ Department of Environmental Sciences, Emory University, Atlanta, GA 30322; ^b^School of the Environment, Washington State University, Vancouver, WA 98686; ^c^ Department of Geography, Portland State University, Portland, OR 97201; ^d^ Department of Biology, Georgia State University, Atlanta, GA 30302; ^e^ Department of Biology and Environmental Studies, Reed College, Portland, OR 97202

**Keywords:** redlining, gentrification, exclusion, displacement, urban segregation

## Abstract

This study investigated environmental justice by developing and comparing four contemporary urban segregation typologies (persistent exclusivity, persistent disadvantage, recent gentrification, and recent displacement-hosting) with historical Homeowners’ Loan Corporation (HOLC) zones and 104 variables spanning the natural and built environments, pollution, environmental hazards, health, and disability. We applied principal component analysis to the 2010 and 2020 American Community Survey and the US Census data to derive neighborhood typology indices for 342 tracts in Portland, Oregon. Using Spearman’s correlation and end-member comparisons, we assessed relationships among the typologies, HOLC zones, and environmental and health outcomes. HOLC zones predicted persistently exclusive areas in the urban core but not current injustices elsewhere. Persistently exclusive neighborhoods, both within and outside HOLC zones, had more tree canopy, less dense development, lower disaster risks, better preventive care, and fewer diseases than other areas, but more air pollution exposure. Recently gentrified areas had more transit and community resources but worse heat exposure, pollution, and impervious surface coverage and aligned strongly with HOLC C and D zones. Recent displacement-hosting neighborhoods were cleaner and greener but lack transit and community resources, while exhibiting weak health outcomes. Persistently disadvantaged areas, often outside HOLC zones, faced the most severe conditions, including minimal access to nature, aging infrastructure, the highest pollution and hazard exposure, and the worst health outcomes and disability rates. Our study offers insights into the spatial mechanisms of urban inequities and well-being, underscoring the need to look beyond redlining to fully understand and address today’s social and environmental injustices.

## Significance

Historical injustices and racial devaluation have concentrated inequities and privileges in certain urban neighborhoods. Yet, these neighborhoods are not static but dynamic and have changed over time. A nuanced understanding of the changes is crucial for identifying environmental injustices today. This study uses widely available datasets to compare historical Homeowners’ Loan Corporation (HOLC) neighborhood classifications with contemporary patterns of socioeconomic exclusivity, gentrification, disadvantage, and displacement. By linking these spatial patterns to environmental amenities, development conditions, hazards, and health outcomes in Portland, Oregon, we reveal a more complex and dynamic geography of inequities than conventional narratives of redlining suggest. Our study offers an innovative, replicable, and evidence-based approach and insight for advancing more just urban policies and planning across US cities.

## Legacies of Historical Redlining

Systemic inequities persist in human–environment interactions in cities worldwide, despite efforts to remedy these challenges (1, 2). Wealth and legacy effects tied to historical injustices continue to influence urban amenity distribution, including green space, biodiversity, ecosystem services, and outdoor health (3, 4). In the United States, poor access to environmental amenities is documented for low-income, racial minority, and formerly “redlined” neighborhoods (5, 6). Redlining was a racially discriminatory home-ownership investment policy established in the 1930s by the US federal government’s Homeowners’ Loan Corporation (HOLC). The HOLC classified residential areas of 239 US cities in four categories to guide (dis)investment: zone A “Best,” zone B “Desirable,” zone C “Declining,” and zone D “Hazardous or Risky.” The zone-D areas, termed “redlined,” were colored red on the HOLC maps and were occupied by Blacks, immigrants, and other minoritized groups (7). Residents of zone C and D areas were systematically denied home loans and insurance by the Federal Housing Authority (FHA) and mortgage banks, leading to limited homeownership, fewer opportunities for wealth creation, and greater social instability. In academic literature and public discourse, the HOLC is well understood to have perpetuated multigenerational cycles of poverty and urban segregation that continue today (5, 8, 9).

Earlier work examining the role of historical HOLC zoning in predicting present-day environmental and community inequities has accelerated societal understanding of how structural factors contribute to persistent urban segregation and environmental injustice (6, 9–13). Many of these studies have linked relative exclusivity in US cities to the availability of natural and built-environment amenities such as tree canopy coverage, green space, and public transit (3–5, 14–16). They have also associated exclusivity with differential exposure to air, water, waste, and noise pollution (10, 17–20), urban heat (21–23), and flood hazards (24). Disproportionately burdened communities of color and low-income residents (11) often experience higher rates of disease and mortality, which are exacerbated by poor access to healthcare (25–27). The availability of clean air, quality affordable housing, potable water, public transit, healthcare, green space, and ecosystem services is shaped by systemic forces such as zoning laws, urban planning, public policy, municipal investment, market dynamics (28, 29), and limited inclusive governance (30). However, whether these patterns mirror those in formerly HOLC-zoned areas across entire cities, and whether such amenities and hazards precede or follow shifts in neighborhood exclusivity, remains less thoroughly examined.

Building on such work, we draw attention to a few key considerations for future studies comparing historical redlining with more recent urban structural and environmental injustice. First, the effects of discriminatory HOLC zoning have not necessarily been uniform or linear between the 1930s and today. Second, the HOLC-zoned areas of 90 y ago are now the small urban cores of today’s cities and so may not exhibit characteristics shared by other neighborhoods or HOLC zones in the past. Third, representing broad, hypothesized patterns of economic, demographic, and environmental segregation using only one or a few variables (e.g., only HOLC zone; income, whiteness; tree canopy, urban heat) risks introducing selection bias (31); statistical results may reflect the specific characteristics of the chosen variables rather than broader community patterns, and the apparent strength of relationships may obscure underlying uncertainty. Fourth, variance, skew, dimensionality, and type mismatches are difficult to address without unintentionally distorting the meaning of the variables. Encoding of categorical data (e.g., HOLC zones) can introduce artificial weight, while methods like logistical regression or ordination may be inappropriate. Skewed variables, such as income, may overemphasize affluence, and standard transformations (e.g., z- or log-scaling) risk misrepresenting the data. Additionally, not all variables are unidirectional, e.g., proximity to transit may have positive or negative health effects depending on context. Finally, redlining and related forms of urban segregation are often framed as binary problems (e.g., affluence vs. poverty, high vs. low asthma rates), overlooking key dimensions like temporal change, intracommunity variation, and confounding factors. One-dimensional framing limits our understanding, encourages zero-sum thinking where gains in one area are seen as losses in another, and narrows the solution space for addressing persistent urban injustice.

To our knowledge, no study has examined urban segregation by both the degree and timing of neighborhood relative structural advantage or disadvantage, while also systematically linking these patterns to comprehensive data on environmental and health inequities. We used data-driven dimensional reduction to create indices of persistent exclusivity, persistent disadvantage, recent gentrification, and displacement-receiving areas, based on census data from the Portland, Oregon region. We then compared neighborhoods identified by these indices with historical HOLC zones in the urban core. To assess environment and health disparities, we analyzed correlations between these neighborhood typologies at the census tract scale and a wide range of publicly available metrics. We demonstrate how structural racism and urban environmental inequity have shifted since the HOLC era, revealing patterns of persistent and evolving injustices. Our conclusion offers deeper insight into the complexity of urban change and highlights the challenges and opportunities in addressing overlapping forms of inequality. Following this introduction is a detailed elaboration of our approaches, methods, analysis, and findings.

## Typologies of Segregated Neighborhoods

Here, we describe contemporary segregated neighborhood typologies in the United States, developed based on existing literature and further quantified in this study.

Persistently Exclusive Areas are characterized by stable mortgages, high home values that support wealth-building, and low-density single-family housing that often restricts multifamily development, thereby limiting affordable housing options (4, 32). These neighborhoods typically have well-developed infrastructure, high-quality schools, and abundant environmental and social amenities (33). Residents are predominantly white, college-educated, and have discretionary income (3, 33). In the United States, such areas have been associated with historical HOLC A and B zones in several studies (4, 13). It remains understudied whether affluence and environmental quality always align in exclusive areas. While the dominant narrative links tree canopy coverage and green spaces with racial and socioeconomic privilege, this pattern does not hold universally. Early studies in newly developed, less-forested cities supported the correlation (e.g., ref. 34), but later research in older, naturally forested cities showed contrasting trends (35, 36), which have yet to be fully integrated into mainstream discourse.

Persistently Disadvantaged Areas are at the opposite end of the exclusivity spectrum, presumed to be lower-income, communities of color, and to have experienced chronic environmental and socioeconomic deprivations through systemic discrimination and exclusion from political processes (37). Whether in urban pockets or rural settings, they are expected to have high poverty and unemployment; limited affordable and accessible healthcare, education, infrastructure, public transit, and broadband internet (38, 39); greater exposure to environmental hazards (e.g., air pollution, floods, and heatwaves) (11, 37); high rates of chronic disease, poor mental health, and shorter life expectancies (8). However, while not discounting that structurally disadvantaged communities are often also disinvested environments, perpetuating a *presumptive* link between neighborhood disadvantage and poor resident health and environmental quality as a prevailing narrative may depreciate the agency of residents, obscure the complexity of intracommunity variations and contributing factors, and limit imaginative solutions.

Recently Gentrified Areas are shifting toward greater exclusivity due to significant public or private investment in urban renewal/redevelopment. These investments often bring enhanced housing, amenities, economic opportunities, and rising homeownership rates, which attract a whiter, more highly educated, and higher-income population (40). However, the process frequently displaces lower-income, long-term residents, particularly communities of color. In the United States, gentrification often occurs in former HOLC C and D zones (41–44). In some cases, increases in property values, displacement, and exclusivity are closely linked to environmental investments (e.g., tree planting and park creation), and so termed “green gentrification” (28, 42, 45). However, not all green investment causes gentrification (46). Development may also drive gentrification while removing trees by attracting new investments and businesses (38, 40). Greening initiatives can precede gentrification by attracting later investments (16, 45, 47). These mixed patterns highlight the need for more systematic research on causal relationships between environmental investment and neighborhood change.

Displacement-hosting Areas reflect the inverse of the gentrification typology, with recently decreased average income, degree attainment, homeownership, and whiteness (48). Though residents of all socioeconomic and racial identities move into and out of gentrifying (48) and other areas in cities, we focused on neighborhoods absorbing structurally disadvantaged residents, either displaced from gentrifying urban cores (38, 48, 49) or new to the city. These areas offer more affordable housing but may have greater unemployment, lower-performing schools (48), and fewer built amenities. A common conceptual model assumes that increased structural disadvantage will correlate with lower or declining average resident health and environmental amenities; however, this is largely untested for the case of displacement-receiving urban areas.

## Results and Discussion

### Patterns of Socioeconomic and Racial Segregation.

**Fig. 1.**
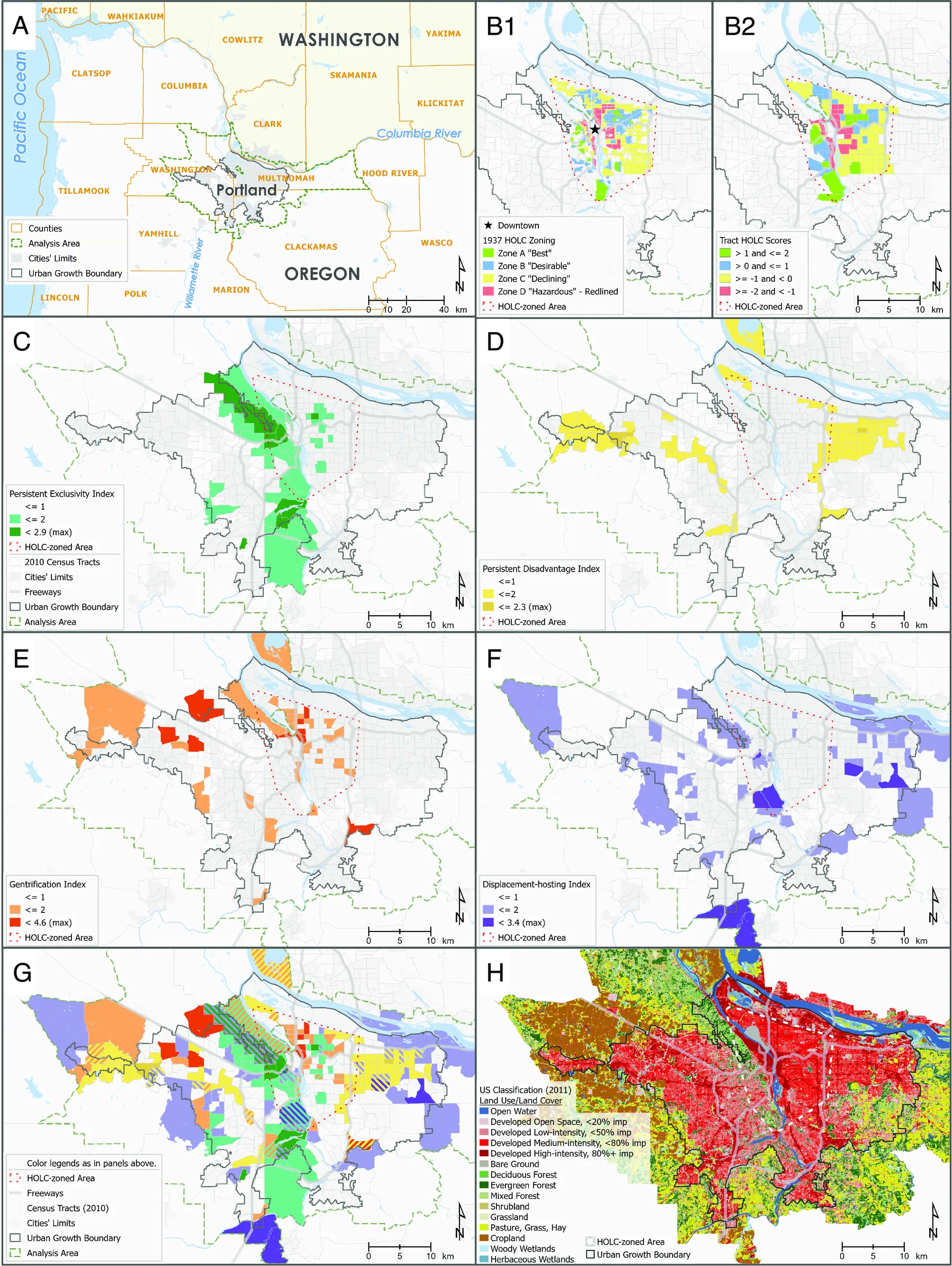
Patterns of historical redlining and neighborhood typologies index. (*A*) Portland, OR, study area. (*B*_1-2_) Historical HOLC zoning numerically scored by tract. (*C*–*G*) Tracts most representative of each neighborhood typology: Persistent Exclusivity, Persistent Disadvantage, Recently Gentrified, Displacement-hosting. (*H*) US national land cover classification.

We derived and encoded the dominant signal of socioeconomic and racial structural privilege across the study region using data-driven dimensional reduction of ACS census inputs. A Persistent Exclusivity Index (PEI) was created as the first principal component (PC1) of census tract data on home value, income, degree attainment, new building, and whiteness in 2010 and 2020. Highly exclusive tracts with PEI values more than one SD above the mean (PEI > 1) included all former HOLC A zones and were equally likely to occur inside or outside the historically zoned urban core (Fig. 1 *B* and *C* and *SI Appendix*, Table S5). Relative privilege in the urban core was statistically related to HOLC zoning, but due entirely to variance in exclusivity among former A/B zones, whereas relative C/D zoning was not at all predictive (*SI Appendix*, Fig. S5); this cautions against overrelying on the lens of historical redlining to explain contemporary neighborhood inequities of exclusivity or disadvantage.

The other end of the exclusivity spectrum was highlighted by a Persistent Disadvantage Index (PDI), the opposite of the PEI. Persistently highly disadvantaged areas (PDI > 1) were not at all predicted by historical HOLC zoning. Instead, these chronically depressed neighborhoods were predominantly (87%; *SI Appendix*, Table S5) outside the historical urban core (Fig. 1*D*). These areas were established when redevelopment of the 1960s+ displaced low-income and communities of color from core downtown and inner-north neighborhoods (14, 50) to north and east Portland (51). We quantified and showed that this previous redevelopment wave gentrified 20% of the redlined (D-zoned) areas of the urban core, which are now persistently highly exclusive (*SI Appendix*, Table S5). Meanwhile, building age data corroborated that these formerly displacement-hosting, now persistently disadvantaged, neighborhoods were built in 1970 on average, only somewhat lagging the average of 1960 in formerly privileged or gentrified, now highly exclusive neighborhoods.

Recent gentrification has converted an additional 45% of redlined (D-zoned), 26% of C-zoned, and 32% of B-zoned neighborhoods in the urban core toward greater exclusivity, consistent with other studies showing gentrification concentrated in HOLC-disenfranchized areas (41–44). No A-zoned areas have gentrified, consistent with privilege resisting redevelopment (*SI Appendix*, Table S5). A Gentrification Index (GFI) was encoded by the PC1 of increased income, degree attainment, whiteness, and new buildings from 2010 to 2020 (*SI Appendix*, Fig. S3). Pronounced recent gentrification (GFI > 1, Fig. 1*E*) focused on the inner east side and in Albina, the north-Portland historical center of the Black community (14, 50), consistent with urban renewal areas (51). Of the four neighborhood typologies, gentrification was the most concentrated within the urban core (59%, *SI Appendix*, Table S6), but has also been expanding outward from persistently highly exclusive areas (Fig. 1*G*). Recent far-west side gentrification on less developed lands (Fig. 1*H*; 52) may reflect amenity migration (53).

Market forces are now driving displacement into new periurban locations near the urban growth boundary and new intraurban pockets (Fig. 1*F*). A Displacement-hosting Index (DHI) was derived as the PC1 of decreased income, degree attainment, whiteness, increased renting, and home values, and non-English-speaking population between 2010 and 2020 (*SI Appendix*, Fig. S4). While the concentration of high DHI (>1) at the urban fringe was anticipated (51), it remains unclear why some new displacement-hosting pockets are emerging near major natural area parks such as Forest Park, Marquam, Tryon Creek, Powell Butte, Kelly Butte, Hogan Butte, and Blue Lake. These urban and periurban communities face an uncertain future, as they exhibit a combination of declining average incomes and rising median home values. Questions about whether they will remain disadvantaged or whether home values will increase in a new wave of gentrification are unclear.

### Emerging Spatial Patterns of Environment and Health Disparities.

**Fig. 2.**
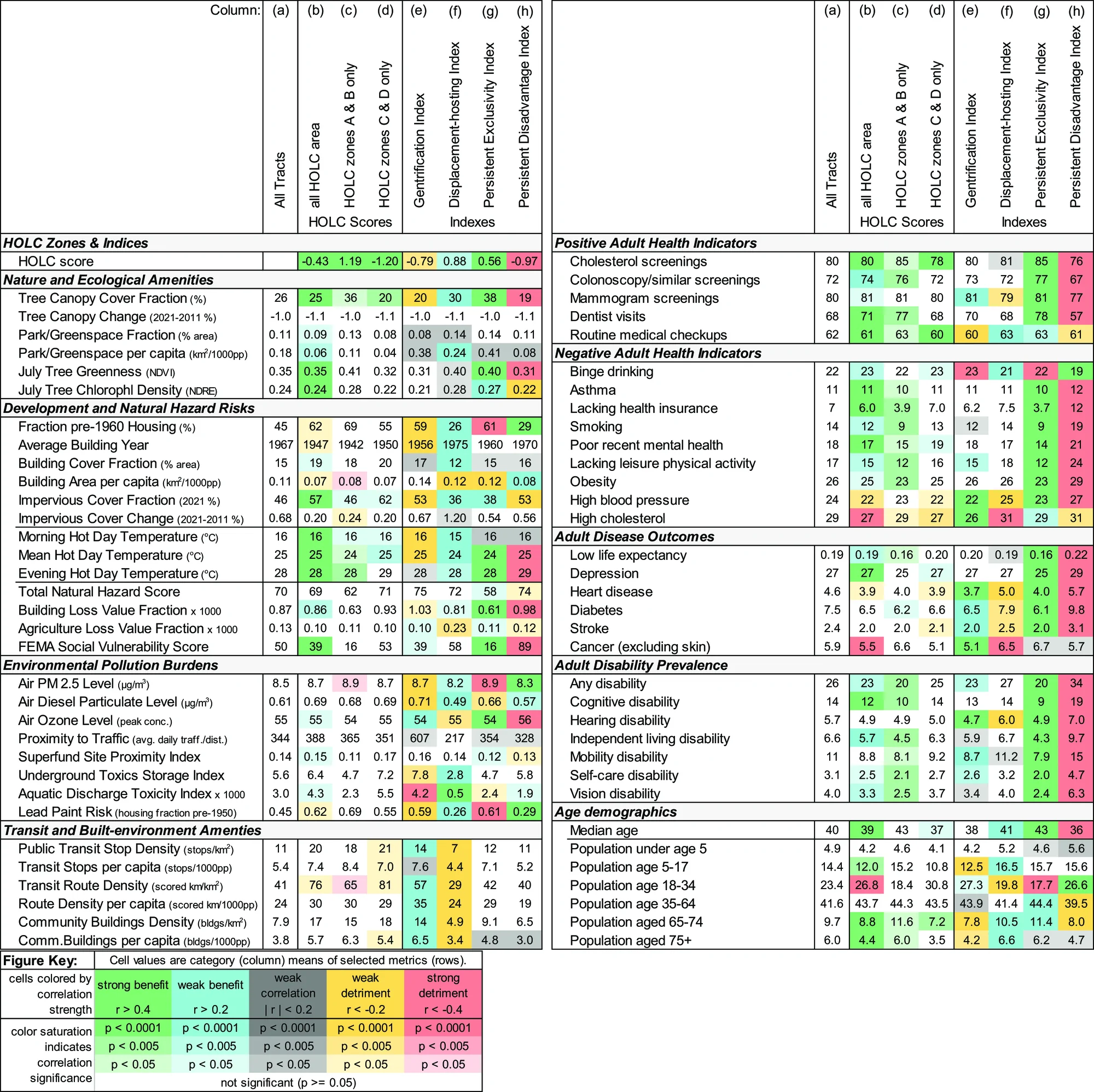
Relations between neighborhood typology indexes, HOLC zoning, and metrics of neighborhood environmental and health conditions. Numbers shown are mean values of selected environment, development, hazard, or health metrics (rows) tabulated for the subset of tracts within the category indicated in each column. For the Indices columns, means were calculated only for the subset of tracts with high values (>1) for that index. Color-coding of cells is explained by the key, with green/blue colors indicating a beneficial Spearman rank-correlation between the environment or health metric (green stronger correlation than blue) and the HOLC or Index score (e.g., more tree canopy or less pollution are beneficial) and yellow/red colors indicating detrimental correlations (red stronger correlation than yellow). The saturation of each color indicates the statistical significance of such correlations.

**Fig. 3.**
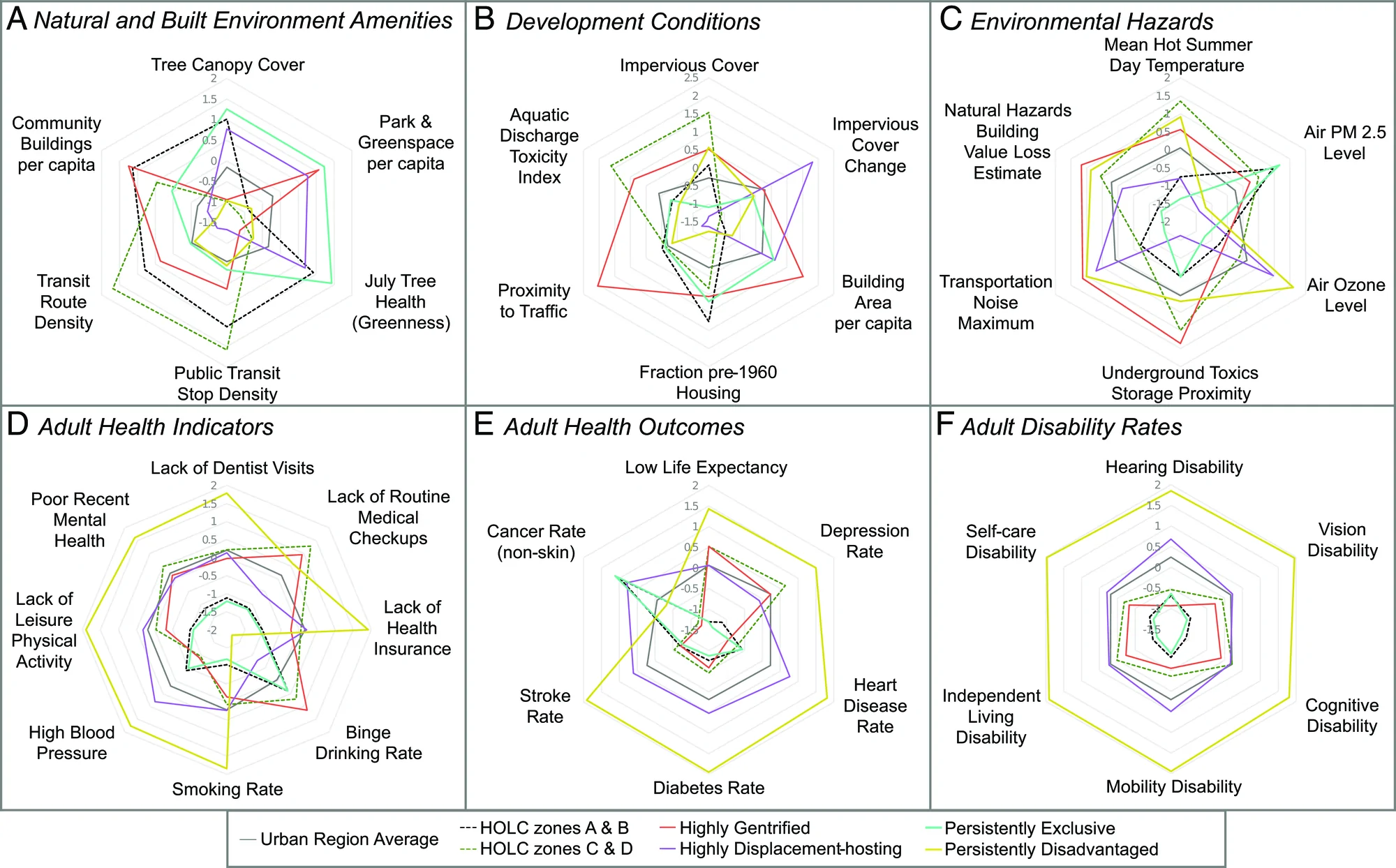
Neighborhood disparities of environmental and community health and amenities. Polygon vertices represent the relative mean magnitudes of the listed (*A*) ecological and built-environment amenities, (*B* and *C*) development conditions and environmental hazards, and (*D*–*F*) health indicators within the subsets of tracts in each type of HOLC Zone or scoring highly on each neighborhood typology index (index > 1). All-tract means in gray. A vertex closer to the outer edge of the background hexagon in (*A*) represents more abundant beneficial natural-built environment amenities; a vertex closer to the outer edge in (*C*–*F*) represents more detrimental environmental or health conditions.

To understand the environmental and health context of neighborhoods, we compared the four contemporary segregation typologies (Index values) and historical HOLC zoning with 104 environmental and health metrics, standardized at the census tract level using z-scores. Drawing from diverse national and local data sources, the analysis included indicators across natural amenities, development and hazards, pollution exposure, preventive health behaviors, health outcomes, disability prevalence, and age demographics. An intentionally broad set of metrics was used to reduce selection bias and then, for clarity, reduced to 67 and 38 representative indicators in Figs. 2 and 3. The results revealed complex and sometimes unexpected tradeoffs among socioeconomic development, demographic shifts, environmental conditions, and health disparities.

### HOLC Discrimination and Today’s Communities.

Neighborhoods historically privileged by the HOLC enjoyed more tree canopy coverage, less heat exposure, and more positive health indicators, on average, in Portland today [green in column (b) of Fig. 2]. However, supposed correlations with HOLC zoning overall were more strongly driven by systematic variance in A and B zones than by C and D zones [e.g., green in column (c) but not column (d), Fig. 2]. In other words, the distinction between Portland’s former HOLC C and D zones is relatively inconsequential for explaining community environment and health conditions today, and the supposed predictability of these factors based on HOLC zoning is driven almost entirely by relative degrees of privilege rather than a contrast between privilege and disadvantage. This finding exemplifies the challenge of underappreciated skew within data, potentially skewing the narrative conclusions if not inspected closely. For metrics of building density, natural hazard and pollution exposures, transit and community resource availability, and most adult disease outcomes, HOLC zoning was only weakly or insignificantly predictive [light blue, yellow, white in column (b), Fig. 2]. For these types of environmental and health metrics, the shared location of all HOLC-zoned neighborhoods within the urban core was the overriding factor, suggesting a need for strong caution if seeking to analytically or narratively extrapolate from variance among HOLC zones to understand or map the environmental and health contexts of exclusivity or disadvantages city-wide.

### Natural Environment and Health Inequities.

Across the region, many beneficial environmental amenities and better resident health accrued to persistently exclusive neighborhoods, while persistent disadvantage correlated with disamenities and morbidity. For example, persistently highly exclusive census tracts had significantly more tree canopy cover (twofold) than highly disadvantaged areas [values in columns (g), (h), Fig. 2]; this aligns with existing studies that suggest exclusive areas (15) may reflect luxury effects (13). Tree canopies moderate urban heat, reduce air pollution, and mitigate environmental conditions that impact health outcomes (54–57). Overall, greater exclusivity was correlated with greater tree canopy and greenness, less impervious cover, lower summer temperatures, lower natural disaster risk, lower ozone levels, and lower rates of detrimental health indicators or outcomes [dark green/blue in column (g), Fig. 2] except binge drinking (reversed) and cancer (not significant). Neighborhoods at the opposite end of the exclusivity/disadvantage spectrum exhibited the opposite trends [column (h), Fig. 2]. However, the more exclusive a neighborhood, the older its housing and higher its lead paint risk, the higher its PM 2.5 and diesel particulate air pollution exposures, and the higher its binge drinking rates [red/yellow, column (g), Fig. 2]. This suggests that benefits from persistent privilege and affluence are sufficient to overcome the potential of detrimental impacts from at least some types of poor environmental quality in this study area, even as exclusivity and better environmental quality and health are generally mutually reinforcing.

Comparing the most highly disadvantaged neighborhoods to highly exclusive, gentrified, or displacement-hosting ones illustrated the starkly worse environmental and health contexts associated with persistent structural disadvantage in our study region (Fig. 3). The greatest disparities were in disability rates, with persistently highly disadvantaged communities showing 70% greater occurrence of disabilities than highly exclusive areas (Fig. 3*F*). These communities also exhibited greater heat and natural hazard risks, suggesting a particular need for equitably accessible hazard mitigation and evacuation plans. and categorically worse health indicators and outcomes despite hosting a younger population (Figs. 2 and 3 *C*–*E*), raising concerns about their future. Although greater persistent disadvantage also correlated with less severe particulate air pollution and lead paint risks in our data [green/blue, column (h), Fig. 2].

Contrasts in the proportion of residents with health insurance were likely also key to the greater likelihood of poor health and disease in persistently more disadvantaged neighborhoods. In persistently highly disadvantaged neighborhoods, 12% of residents lacked health insurance in 2020, compared to only 3.7% in persistently highly exclusive neighborhoods (Fig. 2). With the aid of our mapped indices, we were able to diagnose that this insurance gap persisted in 2020, despite the Affordable Care Act greatly reducing the number of uninsured over the preceding decade, precisely because of this concentration and persistence of neighborhood disadvantage. Analyzing additional census data (58), we found that the majority of tracts with high uninsurance rates in 2013 that persisted with high uninsurance rates in 2020 were almost entirely aligned with our identified persistently disadvantaged neighborhoods (*SI Appendix*, Fig. S8), but tracts that improved insurance rates by 2020 were predominately in “average” areas of our study region (white in Fig. 1*E*). Although insurance is a contributing factor, differential clinic access and transportation, and barriers of language, culture, racism, and classism in medical settings, likely also contribute to health inequities disproportionately burdening these persistently disadvantaged neighborhoods. Gentrified and displacement-hosting areas had regionally average health insurance rates and nonsignificant or inconsistent relationships between health metrics and the degree of recent socioeconomic change (not significant gray/white or mixed yellow/blue, Fig. 2).

### Emerging Intersections of Segregation, Environment, and Health.

Whereas disparities in greenery, natural hazard exposure, and resident health were predicted by *persistent* exclusivity and disadvantage, contemporary disparities in development, pollution, transit, and community resources were more consistently predicted by the *change* in relative exclusivity recently occurring [dark colors in columns (e-f) vs. light/white in columns (g-h), Fig. 2]. Surprisingly, despite pronounced recent decreases in socioeconomic advantage, displacement-hosting areas exhibited among the lowest pollution burdens [blue/green, column (f), Fig. 2] and a more exclusive-like package of environmental amenities (Fig. 3*A*). However, their locations in the more forested and floodplain urban fringes (Fig. 1*F*) provide significantly less access to community resources and transportation. This agrees with a trend of low-income residents and people of color being displaced to greener yet socially fragmented neighborhoods at the urban fringe (59, 60), where slow property appreciation and economic instability may reinforce cycles of poverty and isolation (61, 62). Still, the magnitude of the recent increase in disadvantage in these areas was not significantly related to most health and disability indicators [white/gray, column (f), Fig. 2] and less strongly predicted negative health outcomes than persistently disadvantaged [yellow vs. red, columns (f/h)]. Therefore, we find no evidence that the growing disadvantage in displacement-hosting areas will inevitably lead to the same environmental and health burdens seen in persistently disadvantaged neighborhoods. Thus, indicating that not all disadvantaged areas experience inequality in the same way.

In contrast, despite their increasing privilege, more intensely gentrifying areas were correlated with stronger environmental *dis*amenities, including less tree canopy cover, greater imperviousness, hotter air temperatures, and more pollution exposure [yellow/red, column (e), Fig. 2]. This is attributable to recent gentrification concentrating in core urban areas, e.g., nearer transit but also nearer its pollution and noise (Fig. 3). Redevelopment and green local urban policies have failed to reduce local heat and pollution or increase tree canopy despite substantial recent investment, but our findings are consistent with the idea that an urban growth boundary policy contains both development and heat and other anthropogenic influences (63). Also, as net tree canopy cover change in recently gentrified areas was negligible over the study decade, legal protections for trees may have successfully buffered against development-induced tree loss (64, 65). That more intensely gentrifying areas also exhibited better health outcomes [green/blue, column (e)] despite worse environmental conditions likely demonstrates the ability of increasing affluence to mitigate local environmental detriment, an option not available to the persistently disadvantaged areas that suffer both structural disadvantage and poorer environment and health. Alternatively, this may reflect a lag effect, where recent socioeconomic improvement might more quickly address individuals’ health but may take longer to improve the shared local environment. These results suggest that factors linking socioeconomic and racial neighborhood characteristics to health in rapidly changing neighborhoods differ fundamentally, and substantially, from those in persistently exclusive and disadvantaged areas, possibly because environmental quality trends have run counter to socioeconomic shifts.

### Limits of Redlining as a Predictor of Modern Urban Landscapes.

Our analysis reveals that using redlining alone to interpret contemporary urban landscapes risks reinforcing historical biases, highlighting persistent exclusivity while obscuring complexities in disadvantaged areas and neglecting ongoing timescales of change. A redlining-focused approach will be limited because the historical HOLC maps cover a small area, creating sampling bias that does not extend to the larger, modern urban landscape. Also, the 90-y gap since redlining obscures the effects of intervening redevelopment and expansion. Furthermore, structural privilege is conveyed by many factors other than HOLC zoning that may be equally important, and the greater variance among contemporary privilege compared to the relatively compressed conditions of disadvantage means that socioeconomic analyses may be driven more by variations in privilege, i.e., the quantitative biases favor persistently exclusive areas but not persistently disadvantaged areas. In the Portland area, historical redlining partially explained current urban environmental and health disparities but underestimated the contrasts between privileged and disadvantaged neighborhoods compared to trends across the metro area.

A key theme in our results is that structural legacies of racism and segregated land use predict better environmental quality and health for affluent, white communities, but do not fully capture conditions for less privileged groups without accounting for recent development, displacement, and urban expansion. We find that privilege and stability are mutually reinforcing, as historically privileged areas remain exclusive, while it is less predictable whether or when historically disinvested, redlined areas may remain disadvantaged or be redeveloped and gentrified. Yet, we find that intense urban reinvestment, such as with gentrification, even with a strong greening mission, is not necessarily sufficient to reverse the poorer environmental quality, sparser canopy, and greater pollution burdens of formerly disenfranchised communities of the urban core, at least within a decade or so. Concurrently, when displaced disadvantaged communities are pushed to resource-limited urban fringes, they may enjoy *greater* environmental amenities and slightly better health, though fewer transit and community resources, but may compete for space with privileged amenity migration. These results challenge the common assumption that disadvantaged communities universally face poorer environmental conditions and raise questions about whether future urban policies will accommodate the sometimes contrasting nature of environmental and socioeconomic privileges or create a lasting divide between them.

Overall, the inability of HOLC zoning to predict how socioeconomic changes align with ecological amenities, disease burdens, and community health across the broader urban area highlights the need to look beyond redlining—spatially, temporally, and narratively—to better understand and address contemporary urban inequities. Separating persistent exclusivity and disadvantage from recent gentrification and displacement, such as by our neighborhood typology indices, is a useful approach. The findings from this study not only offer valuable insights for planning a more equitable urban ecology but also highlight the need for further research to identify where policy changes, targeted investments, and resources can create disparity or allow for meaningful improvement in both the environment and human health.

## Methods

### Deriving Neighborhood Typology Indices.

Principal component analysis (PCA) was conducted on selected US Census and ACS data (66) race and socioeconomic variables from 2010 and 2020 (Persistent Exclusivity Index, PEI) or the difference between 2010 and 2020 (GFI and DHI) and housing property value data (67) for the 342 census tracts (68) intersecting the Portland METRO regional planning authority region (69), after inputs’ z-score normalization. Each of the three sets of input variables was based on other recent gentrification, displacement, disadvantage, and vulnerability studies (70–77; also see *SI Appendix*). With this entirely data-driven approach (78), PCA dimensional reduction captured the dominant trend shared among each set of input variables as its first principal component (PC1). For example, to derive the PEI we extracted tract-level ACS data on home value, income, degree attainment, new building, and proportion of white population for 2010 and 2020, z-transformed each of these attributes, input the set of them to PCA, took the resulting tract-level PC1 scores, z-transformed that set of scores, and used the resulting values as our PEI values. This automated, data-mining PEI derivation explained 58% of the total variance among the multidimensional tract-level input, and roughly equally represented all the contributing variables except new building fraction (*SI Appendix*, Fig. S2). Our GFI was derived from PCA of increased household income, degree attainment, white population proportion, and new building development from 2010 to 2020. The GFI was strongly loaded by income and degree attainment and less so by building development and racial shifts (*SI Appendix*, Fig. S3). Displacement-hosting neighborhoods were identified via a PCI-derived DHI from PCA of decreased average household income, degree attainment, and whiteness, and increased renter population, rent, and home values, and non-English-speaking residents between 2010 and 2020. Income and education contributed most strongly to the DHI, followed by home value, race, and language (*SI Appendix*, Fig. S4).

A fifth index was developed to quantify the relative degree of privilege assigned by the HOLC in 1939. As the HOLC zones (79) crossed the boundaries of 2010 census tracts, the HOLC zones also had to be translated to the tract scale. After minor geospatial repair (*SI Appendix*), the zones of the digitized HOLC map were assigned values using a z-score-like range: A = 2, B = 1, C = −1, D = −2. For tracts at least 25% overlain by HOLC zones, the area-weighted mean HOLC score was calculated based on the overlain (not total tract) area. Areas blank, unzoned (including “Industrial”), or not within the original HOLC map extent were not assigned an HOLC score. Of the 342 tracts in the study region, 97 were thus assigned tract-level HOLC scores (Fig. 1*B*_2_).

### Data and Analysis of Environment and Health Metrics.

Environment and health conditions were quantified for each census tract by 104 metrics obtained as GIS or remote sensing data from publicly accessible sources: CAPA Strategies, CDC, City of Portland, DOT, EPA, FEMA, METRO, NLCD, PlanetLabs, and US Census (80–;89). (See *SI Appendix* for all metrics, sources, and preprocessing.) An intentionally broad suite of diverse types of metrics from many sources was employed to mitigate potential selection bias. We aligned the sign of each metric so higher values indicated beneficial conditions, such as tree canopy cover being given a positive sign, but smoking rate a negative sign. We used Spearman’s nonparametric correlation to test the degree to which conditions in neighborhoods exemplifying one of the four neighborhood typologies or HOLC zone types were different from conditions in areas not showing, or showing the opposite, typology. We focused on results with correlation magnitude >0.4, and below a very low threshold for two-tailed significance (*P* < 0.0001), to account for the potential statistical issue of multiple hypothesis testing; however, we also illustrated correlations of only magnitude >0.2 and significance thresholds of 0.005 and 0.05 to inform our findings where stronger correlations were absent (Fig. 2). We intentionally chose nonparametric correlation analysis at the tract level over spatial model analysis to avoid diminishing the contribution of any tract to the overall range and variance represented in the region or to tendencies for neighborhood-by-neighborhood contrasts in socioeconomic, race, health, and environmental factors. To test for differences in environment and health between neighborhood typologies, we extracted the tracts most characteristic of each of the four indices (indices > 1, Fig. 1) or of high or low HOLC-designated privilege (HOLC scores > 0.75 or < −0.75), and calculated the mean value of each environmental or health metric within each of these six subsets, within the overall HOLC-zoned area, and as a region-wide mean. We reported the values of these eight means per metric in Fig. 2. We then transformed each set of eight means to within-set z-scores to normalize their scales for display in Fig. 3.

See *SI Appendix* for further details on methods and results.

## Supplementary Material

Appendix 01 (PDF)

## Data Availability

All study data are included in the article and/or *SI Appendix*.
