# Supplementary material for: Beyond redlining: Gentrification, displacement, disadvantages, and exclusivity predict urban environmental and health inequities
Source: Proc Natl Acad Sci U S A. 2026 Sep 14;123(38):e2422760122. doi: 10.1073/pnas.2422760122 (PMC13598229; doi:10.1073/pnas.2422760122)
Supplement: Supplementary file 1 [file pnas.2422760122.sapp.pdf]

PNAS

Supporting Information for

Beyond Redlining: Neighborhood Gentrification, Displacement, Disadvantages, and Exclusivity Predict Urban Environmental and Health Inequities

Idowu Ajibade*, Kevan B. Moffett*, Jason Maxfield, Kate Gregory, Axcelle Bell, Jackson Voelkel, Todd Rosenstiel, Aaron R. Ramirez

*Authors contributed equally

Corresponding author: Idowu Ajibade

Email: iajbad@emory.edu

SUPPORTING INFORMATION TABLE OF CONTENTS

SUPPORTING INFORMATION: METHODS 3

A. Development of neighborhood typology indices ***3***

a. Socioeconomic variable selection 3

Table S1. ACS variables and references used to inform Indexes 3

Figure S1. Schematic overview of data preparation and analysis 4

b. Socioeconomic data sources 5

c. Data pre-processing 6

Table S2. Input variables calculated for use in deriving neighborhood indices 7

d. Index development by Principal Components Analysis 8

i. Conceptual basis for PCA approach to deriving indices 8

ii. PCA computation details 8

B. HOLC map processing ***9***

a. HOLC map digitization repairs 9

b. HOLC zone numerical encoding 9

C. Neighborhood characteristics of environment and human health ***10***

a. Environment and health data and sources 10

Table S3. Environment and health metrics and data sources 11

b. Environment and health data pre-processing 14

i. Street Tree and Park Tree Inventory data 14

ii. Parks and Greenspace, Rural Reserves, Urban Reserves 14

iii. Tree canopy greenness and chlorophyl density 16

iv. Transportation Noise 16

v. Building footprints, area, and density 16

vi. Estimated air temperatures 17

vii. Transit Stations 17

viii. Transit Routes 17

ix. Community Buildings 19

c. Neighborhood Index and HOLC relations to environment and health data 19

SUPPORTING INFORMATION: RESULTS 20

D. Results of neighborhood indexes' derivations ***20***

Table S4. ACS attributes' PCA loadings on each neighborhood typology index 20

a. Persistently Exclusive Areas PCA results 21

b. Gentrified Areas PCA results 21

c. Displacement-hosting Areas PCA results 21

Figure S2. Persistent Exclusivity Index PCA derivation results 22

Figure S3. Recent Gentrification Index PCA derivation results 23

E. Relations of HOLC zones and indices ***25***

Table S5. Relations between neighborhood typologies and HOLC zones 25

Figure S5. Relations of neighborhood indices to HOLC scores 26

Table S6. Contemporary fates of historical HOLC zones 28

Table S7. Apparent origins of contemporary neighborhood typologies among HOLC zones 28

F. Spotlight on health insurance rates ***29***

Table S8. Proportion of population without health insurance, 2010 and 2020 29

Figure S6. Portland-area Census Tracts map of percent uninsured in 2013. 30

Figure S7. Portland-area Census Tracts map of percent uninsured in 2020. 30

Figure S8. Persistently highly disadvantaged areas also most persistently lack medical insurance between 2013 and 2020. 31

REFERENCES 32

SUPPORTING INFORMATION: METHODS

A. Development of neighborhood typology indices

a. Socioeconomic variable selection

As the first step of Index development, we extracted three different, specific, and intentional datasets from US Census American Community Survey (ACS) source data for 2010 and 2020, to inform the three indices (1). Different source variables were needed to represent the key neighborhood characteristics of persistent exclusivity/disadvantage, recent gentrification, or recent receipt of displaced individuals because of the different demographic and socioeconomic factors, processes, and directions of change inherent in each of these categories. The three source datasets were selected based on the findings of other recent gentrification, displacement, and vulnerability modeling studies (Table S1).

An overview of the study methodology is provided in Figure S1 (next page).

Exclusivity and Disadvantage attributes:

To capture exclusive areas, we chose variables that represent privileged demographic groups, as well as housing related variables (Table S1). The housing variables included property value, to capture areas that are harder to move into because of higher values, and percentage of buildings built before 2010 because of the older housing stock that characterizes exclusive areas.

Gentrification attributes:

We created the gentrification index to capture both the outflow of marginalized populations and the influx of privileged populations, along with changing infrastructure and increasing housing values that are characteristic of gentrifying neighborhoods (2–4).

Displacement-hosting area attributes:

The displacement-hosting index captured areas represented by an influx of marginalized demographic groups over the 10 year period. These methods build on work by Ding et al. (5), who found that residents displaced from gentrifying areas were more likely to move to lower income neighborhoods. We selected five demographic variables (% population without a bachelor’s degree, % population of color, % renter occupied households, % population with below median household income, % limited English speaking population) as well as median housing and median rental values.

Table S1. ACS variables and references used to inform Indexes

Variable Type	Indexes			References									
	Exclusivity / Disadvantage	Gentrification	Displacement-hosting	(2)	(4)	(6)	(7)	(8)	(9)	(10)	(11)	(12)	(13)
Educational Attainment	X	X	X	x		x	x	x					
Racial / Ethnicity	X	X	X	x		x	x	x	x	x	x		
Income	X	X	X			x	x	x	x	x	x		
Renters and Rent Values			X						x	x	x		
Language			X									x	x
Property Values	X	X	X			x	x			x			
Housing Type	X	X			x			x					
Housing Stock	X	X						x					

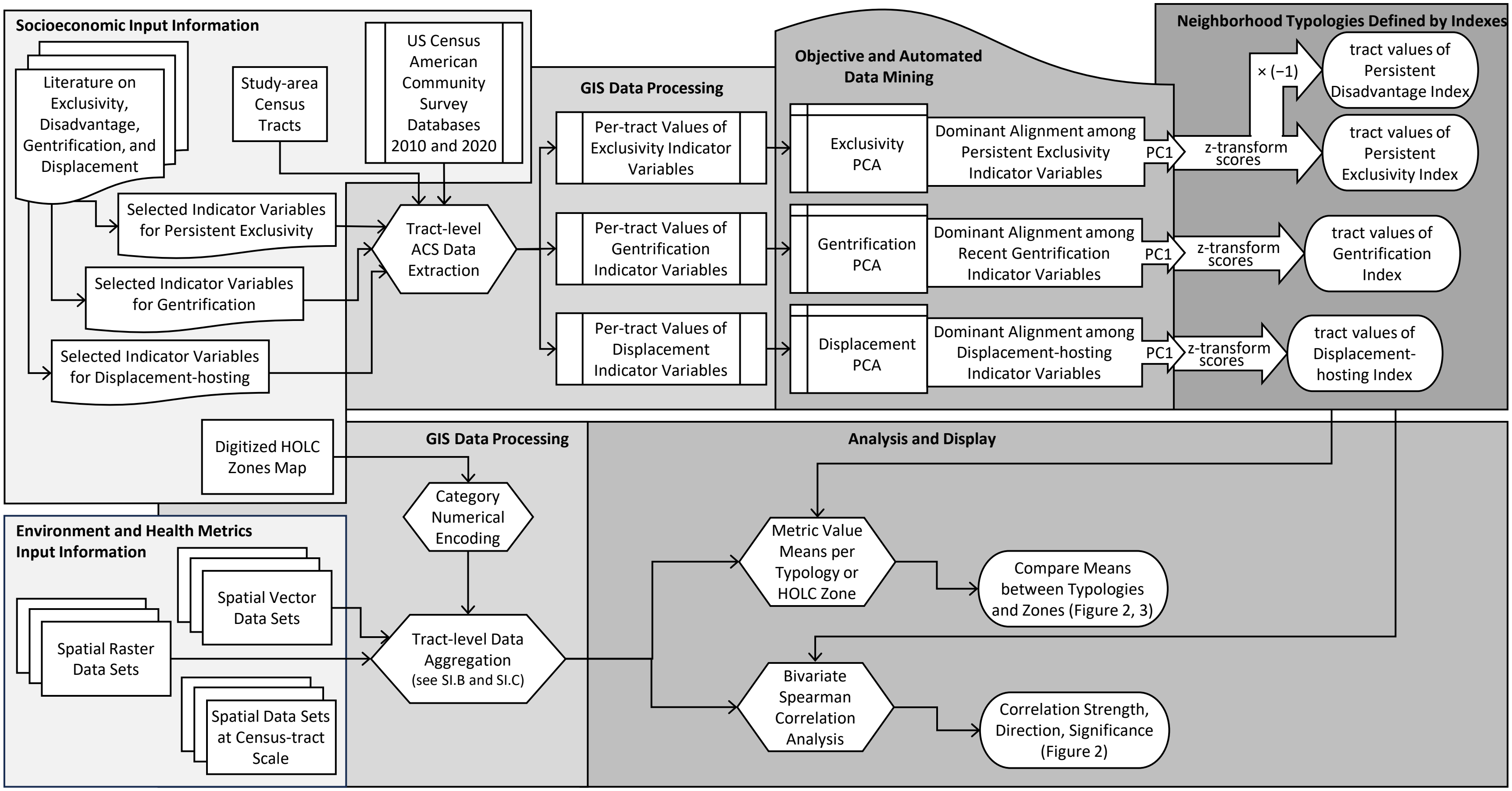

Figure S1. Schematic overview of data preparation and analysis

b. Socioeconomic data sources

The data used in deriving the socioeconomic neighborhood typology indices were obtained from US Census American Community Survey (ACS) data for 2010 and 2020 or the nearest available year (1)

The specific tables used were the following.

- Educational Attainment:
    - Table B15003: from 2012 and 2022 ACS 5-Year Estimates Detailed Tables
    - To calculate percent of tract population with a bachelor’s degree in 2012 (or 2022), the four values for number of people with a bachelor’s degree and higher degrees were summed, then divided by the total population listed for the tract.
    - Percent of population without a bachelor’s degree was calculated as 100% - percent with a bachelor’s degree.
- Housing Tenure:
    - Table B25003: (2010 and 2020)
- Race:
    - Table P1: (2010 Decennial Census and 2020 Decennial Census)
- Income:
    - Table B19013: (Median) (2010 and 2020)
    - Table B19001: (Income distribution) (2010 and 2020)
- Language:
    - Table B16001 (2010 and 2020)
- Home Value:
    - Table B25077 (2010 and 2020)
- Rent Value:
    - Table B25064 (2010 and 2020)
- Year Structure Built:
    - Table B25034 (2022)
    - To calculate percent of buildings built 2010-2019 vs before 2010:
        - Use table from 2022 (only table available at the time of the analysis).
        - The total number of buildings listed in the table is from 2022, so recalculate the total as of 2020 by subtracting the listed value for “built 2020 to present” from the listed value of “total” (2022.
        - Take listed value for number of buildings built 2010-2019 and divide by this total as of 2020 = percent of buildings built 2010-2019.
        - Calculate: 1 - (percent of buildings built 2010-2019) = percent buildings pre-2010.

An example procedure to find and download the relevant ACS data tables:

- ➔ For attribute: percent of tract population with a bachelor’s degree
    - advanced search at data.census.gov
        - click: Geography > Census Tract > Oregon >
            - > Multnomah County > click top box to select “All Census Tracts” in county then click back arrow (<)) above tracts list, then repeat for:
            - > Clackamas County > select “All Census Tracts”
            - > Washington County > select “All Census Tracts”
            - after selecting all three counties’ tracts in the TriCounty area in this way…

- ◆ type: B15003 in the search bar at the top, and press Enter
  - click on "View All" under Tables
  - click on "2012 ACS 5-Year Estimates Detailed Tables" (don't worry if it says table is too big to show)
  - click on "More Tools" at the top-right of the table, then Download, as Excel or csv
- ◆ repeat for "2022 ACS 5-Year Estimates Detailed Tables
- ◆ then follow post-processing calculations above to arrive at final attribute values used

c. Data pre-processing

We pre-processed the raw ACS data in two ways: first, to capture the persistence of more socioeconomically exclusive or disadvantaged characteristics; second, to highlight change toward more exclusive (gentrifying) or more marginalized and disadvantaged community characteristics (displacement-hosting areas). The 2010 Census tracts obtained from METRO (14) were used as the basemap for all analysis.

Exclusivity/Disadvantage attributes:

To capture persistence of community characteristics, we used both the 2010 and 2020 values of the extracted ACS data as separate indicator attributes, without calculating the difference between the two decades (Table S2). This is because we were interested in census tracts with consistently high percentages of privileged populations and older, highly valued housing stock in both 2010 and 2020. The ACS variables selected were expected to have positive relations with exclusivity, so the separate retention of values from both years had the effect of highlighting the variable's indication of exclusivity if the values, such as median home value, were high in both 2010 and 2020. In contrast, the contribution of the variable as an indicator of persistent exclusivity was diminished if the tract showed a high value in one year but a low value in the other year, as the opposite indications from the two years effectively canceled out as indicators of persistent exclusivity.

Gentrification attributes:

To capture the community changes characteristic of increasingly gentrifying areas and increasingly displacement-hosting areas we looked at the magnitudes and directions of the differences between the 2010 and 2020 values for the key variables reported by the decadal Census and the ACS. For variables obtained from ACS as percents (e.g., percent of census tract population with a bachelor's degree, percent of population identifying as white, percent of renter-occupied households), the indicating attribute was calculated by directly subtracting the 2010 percent value from the 2020 percent value, yielding an indicator attribute value of percentage-point change over the decade. For variables obtained from ACS as absolute values (median home and rent values), the percent change in the value was calculated as

$$[100\% \times (2020 \text{ value} - 2010 \text{ value})/(2010 \text{ value})]$$

and the result used as the indicator attribute.

Displacement-hosting attributes:

For each demographic variable, we calculated the percentage point difference between 2010 and 2020, as with the gentrification attributes (Table S2).

Finally, to control for different magnitudes and ranges among the input attributes, and to focus on relative values among tracts within the study region, the set of tract-by-tract attribute values intended to inform quantifying each of the three neighborhood typologies was transformed to a set of z-scores for each indicator (15).

In the table below:

- For demographic and housing changes over time related to GFAs or DTAs, variables were calculated so as to have expected positive relations with the indices. Differences

were calculated as percentage point difference between 2010 and 2020 (* below), as percent change between 2010 and 2020 (** below), or other method as indicated below.

- For demographic and housing characteristics related PEAs and PDAs, differences were not in time; instead the distributions of z-scores of each input variable in both 2010 and in 2020 were used as inputs, to enable detecting persistently high (or low) values, such as property values.
- The final line in each box below states in brackets the variable’s [coded name], as shown in the raw statistical results documented later in this document.

Table S2. Input variables calculated for use in deriving neighborhood indices

This table lists the variables derived from ACS data that were input to the PCA data-driven dimensional reduction process to produce the Indices of recent urban Displacement-hosting areas, Gentrified areas, or Persistently Exclusive or Disadvantaged areas. Differences in time were calculated either as percentage point difference between 2010 and 2020 (* below), as percent change between 2010 and 2020 (** below), or using other method as indicated. Raw variable names used in the data analysis scription for this study are indicated in square brackets, to match our raw variable names as listed in raw analysis output shared later in this document.

**Variable Type**	***Input Attributes for:*** **Persistent Exclusivity or Disadvantage Index**	***Input Attributes for:*** **Gentrification Index**	***Input Attributes for:*** **Displacement-hosting Index**
degree attainment	% population with at least a bachelor’s degree in 2010<br>[bach_yes_pct_10]	increase in % population with at least bachelor degree<br>(% in 2020 - % in 2010)*<br>[bach_yes_diff]	increase in % population without a bachelor’s degree<br>(% in 2020 - % in 2010)*<br>[bach_no_diff]
	% population with at least a bachelor’s degree in 2020<br>[bach_yes_pct_20]		
race, language	% population identifying as white-only in 2010<br>[re_nh_caw_pct_10]	increase in % population identifying as white-only<br>(% in 2020 - % in 2010)*<br>[re_nh_caw_diff]	increase in % population not identifying as white-only<br>(% in 2020 - % in 2010)*<br>[poc_diff]
	% population identifying as white-only in 2020<br>[re_nh_caw_pct_20]		increase in % households speaking limited English<br>(% in 2020 - % in 2010)*<br>[language_diff]
renting, housing type		increase in % multi-family housing<br>(% in 2020 - % in 2010)*<br>[ht_attached_diff]	increase in % renter-occupied households<br>(% in 2020 - % in 2010)*<br>[rent_occupied_diff]
income, housing value	% households with above the median tract income in 2010<br>[inc_over_med_pct_10]	increase in % households with above the median tract income<br>(% in 2020 - % in 2010)*<br>[inc_over_med_diff]	increase in % households below the median tract income<br>(% in 2020 - % in 2010)*<br>[income_diff]
	% households with above the median tract income in 2020<br>[inc_over_med_pct_20]		
	median home value in 2010<br>[medval_10]		percent change median home value<br>(2020 value/2010 value – 1) x 100%**<br>[medval_chg]
	median home value in 2020<br>[medval_20]		percent change median rent value<br>(2020 value/2010 value – 1) x 100%**<br>[rentval_chg]
building age	% buildings built before 2010<br>[built_2010pre_pct]	% buildings built in years 2010 through 2019<br>[built_2010plus]	

d. Index development by Principal Components Analysis

For each index, the input attribute z-scores were loaded into a Principal Component Analysis (PCA). We chose PCA based on the use by Messer et al. (16) and because of the ability of PCA to find statistical means of representing the greatest variance between variables instead of attempting to choose weights ourselves. The output for each index has both positive and negative numbers, with higher positive values representing more displacement, gentrification, or exclusivity, and negative values representing less displacement, gentrification, or exclusivity.

i. Conceptual basis for PCA approach to deriving indices

The conceptual premise of this approach is the following.

1. A diverse but succinct set of input attributes is pre-selected, based on expert knowledge at the vanguard of relevant literature, for indicating a primary shared signal that the input attributes have in common and that is of meaningful semantic interest, e.g., the "exclusivity" of a census tract.

2. Each of the selected indicator attributes is quantified by a distribution, its values from all census tracts in the study region, and the sign and magnitude of the indicator attributes' values are hypothesized to relate positively to the signal of interest (e.g., higher tract-median home values positively related to greater exclusivity).

3. The PCA algorithm ingests the indicator attributes' distributions, arithmetically identifies the key axes of numerical co-variation among those distributions (i.e., the principal components), and calculates the sets of weighted partial contributions from each input attribute that best describe those key axes of co-variation (i.e., the attribute loadings).

4. The principle components returned by the PCA represent emergent properties of, and defined by, the set of input attributes and their distributions. The first principal component represents, mathematically, the one, dominant axis of coordinated variation emergent from the selected input attributes' numerical distributions. Semantically, this first principal component represents, by design, that 'primary shared signal that the input attributes have in common and that is of meaningful semantic interest' (from 1, above). For example, PCA thus reduced the information content from the several persistent exclusivity indicator input attributes (Table S2) to one dominant axis (the first principal component) aligned with the extant spectrum of greater-to-lesser exclusivity (lesser-to-greater disadvantage) of the tracts comprising the study region.

ii. PCA computation details

To achieve dimensional reduction on each of the three sets of contributing indicator attributes, and to derive quantitative Indices of Persistent Exclusivity and Disadvantage, recent Gentrification, and increasingly Displacement-hosting areas, we applied Principal Components Analysis (PCA) as by Messer et al. (16) using JMP statistical software (JMP 15.2, SAS Institute, Cary, NC). A separate PCA was performed using each of the three sets of selected indicator attributes (Table S2).

This approach mathematically implements the premise that, as the input attributes to the PCA were specifically selected for being known indicators of a strongly shared phenomenon of interest (e.g., gentrification), the signal of this reigning phenomenon will be represented by the first principal component (PC1) of variation emergent from the collective numerical distributions of the input attributes. These input distributions are defined by the attributes of all census tracts in the study region, and the relative rank of each census tract with respect to the phenomenon of interest is output as the tract's score along the resulting PC1.

To enhance comparability among the scores from the separate PCAs, we transformed the values of each of the three first principal components to z-scores, compressed the range of outliers by reassigning any z-score with an absolute value greater than four to equal four (or negative four), and took the resulting three sets of PC1 z-scores to represent our three key indices, the persistent Exclusivity Index, Gentrification Index, and Displacement-hosting area Index.

A fourth index, the persistent Disadvantage Index, was calculated as the opposite of the Exclusivity Index. The index values were thus designed to be interpretable as direct measures of deviation from the study area mean with respect to the phenomenon of interest: e.g., a tract with

an Exclusivity Index value = 1.0 was one standard deviation ‘more exclusive’ than the study region mean.

B. HOLC map processing

The HOLC redlining maps were originally created in the 1930’s, when the extent of many cities was different than current boundaries and many currently residential areas had not been developed.

a. HOLC map digitization repairs

Minor adjustments had to be made to the digitized HOLC map file obtained for the study (17), as it was inconsistent in treatment of city parks. Some parks were included with the adjacent zoned neighborhood area, while others were excluded, even if (but not always) they were completely contained within a single HOLC rating polygon. The HOLC zone boundaries were slightly amended in the following ways:

1. For parks previously excluded from the HOLC designations but surrounded by an HOLC zone on three or four sides, the park was counted as part of that surrounding zone.

2. For parks spanning a boundary between two HOLC zones such that both zones surrounded part of the park on two or three sides, the park was split along the shortest line connecting the opposing boundary vertices, and the split portions of the park included within its surrounding zone.

3. Parks on the boundary of HOLC-zoned and un-zoned areas were split so as to include the portion of the park surrounded in a concave way by zone boundary in the zone and leave the remainder area of the park as part of the un-zoned area outside the HOLC map extent.

b. HOLC zone numerical encoding

The original HOLC zoning was categorical rather than numerical. The HOLC map for Portland, Oregon consisted of many large polygons representing the rated areas, which did not necessarily align with boundaries of contemporary census tracts, so some census tracts spanned more than one zone category. Census tract boundaries used as the basemap for this study were from 2010, (14). The HOLC-rated areas also did not include all residential areas within current city boundaries.

To account for multiple zones within a tract, and to facilitate comparable analyses with our other data sets, a numerical “HOLC score” was calculated for census tracts that intersected the historical HOLC map using the following methods.

1. A digitized copy of the historical HOLC zoning map was obtained for the study (17), and amended in small areas to treat parks more consistently (see above).

2. HOLC zone polygons were encoded numerically over a z-score-like range as:

$$A = 2, \quad B = 1, \quad C = -1, \quad D = -2.$$

3. As the HOLC zone polygons were not contiguous with modern census tract boundaries, an area-weighted mean HOLC score was calculated for each tract using QGIS (3.32.1-Lima, 2023), based only on the portions of each census tract covered by the historical HOLC map extent.

Areas blank, un-zoned (including marked ‘Industrial’ by the HOLC), or not within the original HOLC map extent were not assigned an HOLC score. Tracts with less than 25% of their area overlapped by an HOLC zone were also not assigned an HOLC score, to prevent misleading ‘bleed’ of the HOLC zoning at the fringes of the historically redlined area.

Of the 342 total census tracts in the study region, 97 were thus assigned tract-level HOLC scores (see manuscript Figure $1b_2$).

C. Neighborhood characteristics of environment and human health

a. Environment and health data and sources

In this study we sought to understand how recent demographic change processes impact current urban residents' access to environmental amenities and ecosystem services, their wellbeing, and how urban environmental (in)equity differs between our four contemporary segregation typologies and the legacy HOLC zonation. To do so, we compared HOLC zoning and our Indices to suite of 104 metrics groups representing a variety of environmental and human health conditions, hazards, and amenities (Table S3). In the table, publishers of the data sources used are both named and their citations numbered (14, 18–26) as per the reference list, for easier transparency.

Within several thematic areas of environmental amenities, environmental pollution and other hazards, human health indicators, and health outcomes, we sought an overall perspective on the relation of the theme to the historical or contemporary patterns of urban socioeconomic segregation, rather than a focus on any one specific variable. As such, we intentionally selected a large and diverse set of environmental and human health variables to comprise each theme, and drew the data for those variables from multiple very different and independent sources. Wherever possible we used standardized data sources that are available with national coverage in the U.S. (e.g., national tree canopy coverage data, national human health data), or types of data commonly available from the geospatial data resources of cities in the U.S. and around the world (e.g., public transit maps, park and building areas).

Table S3. Environment and health metrics and data sources.

Theme	N	Metric	Shown in:		Our Raw Name	Source	Dataset Name	Our Pre-processing	Tract-aggregation
Nature and Ecological Amenities									
	1	Tree Canopy Cover Fraction (%)	Fig. 2	Fig. 3	NCLD_canopymean	USGS (18)	Percent Tree Canopy Cover, 2019	(Downloaded NLCD product was derived from Landsat satellite data with 30m pixels.)	Mean of pixels in tract.
	2	Tree Canopy Change (2021-2011 %)	Fig. 2		Canopy cover change mean		Percent Tree Canopy Cover, 2021 and 2011	Pixel-by-pixel subtraction 2021 data - 2011 data.	
	3	Inventoried Tree Cover Percent			Buffer_percent	City of Portland (19)	Street Tree and Park Tree Inventories	See pre-processing notes section C.b.i, below.	Sum in tract / tract area
	4	Tree Diameter at Breast Hieght			DBHMean				Mean of inventory trees in tract.
	5	Tree Height			TreeHeightMean				
	6	Tree Crown Width			CrownWidthMean				
	7	June Tree Greenness			JnNDVIMean	Planet Labs (20)	Planetscope PSOrtho5b, 20-Jun-2021 and 29-Jul-2021	See pre-processing notes section C.b.iii, below.	
	8	July Tree Greenness (NDVI)	Fig. 2	Fig. 3	JulNDVIMean				
	9	June Tree Chlorophl Density			JnNDREMean				
	10	July Tree Chlorophl Density (NDRE)	Fig. 2		JulNDREMean				
	11	Park/Greenspace Fraction (% area)	Fig. 2		ORCAdens	METRO (14)	Outdoor_Recreation_and_Conservation_Areas & ORCA Sites	See pre-processing notes section C.b.ii, below.	
	12	Park/Greenspace per capita (km^2/1000pp)	Fig. 2	Fig. 3	ORCApp				
	13	Rural Reserve Area Fraction			RResDens		Urban_and_rural_reserves		
	14	Rural Reserve Area Per Capita			RResPP				
Development and Natural Hazard Risks									
	15	Impervious Cover Fraction (2021 %)	Fig. 2	Fig. 3	PctImp2021	USGS (18)	Percent Impervious, 2021	(Downloaded NLCD product was derived from Landsat satellite data with 30m pixels.)	Mean of pixels in tract.
	16	Impervious Cover Fraction (2011 %)			PctImp2011		Percent Impervious, 2011		
	17	Impervious Cover Change (2021-2011 %)	Fig. 2	Fig. 3	PctImp Change Mean		Percent Impervious, 2021 and 2011	Pixel-by-pixel subtraction 2021 data - 2011 data.	
	18	Fraction pre-1960 Housing (%)	Fig. 2	Fig. 3	PRE1960PCT	EPA (21)	(See Raw Name)	(not needed)	Obtained at tract level.
	19	Average Building Year	Fig. 2		BldYearMea	METRO (14)	Building_footprint_database	See pre-processing notes section C.b.v, below.	
	20	Total Building Area			BldAsum				
	21	Building Cover Fraction (% area)	Fig. 2		BldAdens				
	22	Building Area per capita (km^2/1000pp)	Fig. 2	Fig. 3	BldApp				
	23	Urban Reserve Area Fraction			UResDens	METRO (14)	Urban_and_rural_reserves	See pre-processing notes section C.b.ii, below.	
	24	Urban Reserve Area Per Capita			UResPP				
	25	Morning Hot Day Temperature (ºC)	Fig. 2		am_t_f	CAPA (22)	rasters_chw_portland_092823.zip datasets: am_t_f, mean_temp, af_t_f, pm_f_f, mean_hi	See additional information in section C.b.vi, below.	Mean of pixels in tract.
	26	Mean Hot Day Temperature (ºC)	Fig. 2	Fig. 3	mean_temp				
	27	Afternoon Hot Day Temperature (ºC)			af_t_f				
	28	Evening Hot Day Temperature (ºC)	Fig. 2		pm_t_f				
	29	Max. Mean Hot Day Temperature			mean_hi				
	30	Transportation Noise Mean			Noise2020m	DOT (23)	CONUS_rail_road_and_aviation_noise_2020	See pre-processing notes section C.b.iv, below.	Mean of pixels in tract.
	31	Transportation Noise Mean			Noise202_1				
	32	Transportation Noise Maximum		Fig. 3	Noise202_2				
	33	Total Natural Hazard Loss Risk	Fig. 2		EAL_SCORE	FEMA (24)	(See Raw Names)	(not needed)	Obtained at tract level.
	34	Natural Hazard Expected Annual Loss: Total $			EAL_VALT				
	35	Natural Hazard Expected Annual Loss: Buildings $			EAL_VALB				
	36	Natural Hazard Expected Annual Loss: People $			EAL_VALP				
	37	Natural Hazard Expected Annual Loss: Agriculture $			EAL_VALA				
	38	Natural Hazard Annual Loss Rate: Buildings Value Fraction	Fig. 2	Fig. 3	ALR_VALB				
	39	Natural Hazard Annual Loss Rate: People Value Fraction			ALR_VALP				
	40	Natural Hazard Annual Loss Rate: Agriculture Value Fraction	Fig. 2		ALR_VALA				
	41	FEMA Social Vulnerability Score	Fig. 2		SOVI_SCORE				

Table S3 continued on next page.

Table S3, continued (page 2 of 3)

Theme	N	Metric	Shown in:		Our Raw Name	Source	Dataset Name	Pre-processing	Tract-aggregation
Environmental Pollution Burdens									
	42	Air PM 2.5 Level (µg/m^3)	Fig. 2	Fig. 3	PM25	EPA (21)	(See Raw Names)	(not needed)	Obtained at tract level.
	43	Air Diesel Particulate Level (µg/m^3)	Fig. 2		DSLPM				
	44	Air Ozone Level (peak conc.)	Fig. 2	Fig. 3	OZONE				
	45	Air Toxics Cancer Risk			CANCER				
	46	Air Toxics Respiratory Risk			RESP				
	47	Toxic Releases to Air			RSEI_AIR				
	48	Proximity to Traffic (avg. daily traff./dist.)	Fig. 2	Fig. 3	PTRAF				
	49	Superfund Site Proximity Index	Fig. 2		PNPL				
	50	Risk Management Plan-Facility Proximity			PRMP				
	51	Hazardous Wast Proximity			PTSDF				
	52	Underground Toxics Storage Index	Fig. 2	Fig. 3	UST				
	53	Aquatic (Wastewater) Discharge Toxicity Index	Fig. 2	Fig. 3	PWDIS				
	19	Lead Paint Risk (housing fraction pre-1950)	Fig. 2		PRE1960PCT				
Transit and Built-environment Amenties									
	54	Public Transit Stop Density (stops/km^2)	Fig. 2	Fig. 3	TransPtDen 2	METRO (14)	TriMet bus system stops, TriMet Park & Ride lots, Light rail stations, Rail transit stations, TriMet transit centers	See pre-processing notes section C.b.vii, below.	
	55	Transit Stops per capita (stops/1000pp)	Fig. 2		TransPtPP 2				
	56	Transit Route Density (scored km/km^2)	Fig. 2	Fig. 3	TransRteDe 2		Light_rail, TriMet_Bus_System_(routes), Bike_Routes, Sidewalks, Trails	See pre-processing notes section C.b.viii, below.	
	57	Route Density per capita (scored km/1000pp)	Fig. 2		TransRtePP 2				
	58	Community Buildings Density (bldgs/km^2)	Fig. 2		ComBldgP_1	METRO (14)	Community_Centers, Hospitals, Libraries, Fire Stations, City Halls, Schools, School Sites (polygons)	See pre-processing notes section C.b.ix, below.	
	59	Comm.Buildings per capita (bldgs/1000pp)	Fig. 2	Fig. 3	ComBldgP_2				
Positive Adult Health Indicators									
	60	Cervical cancer screening among adult women aged 21-65 years			Cervical cancer screenings	CDC (25)	(See Metric name)	(not needed)	Obtained at tract level.
	61	Cholesterol screening among adults	Fig. 2		Cholesterol screenings				
	62	Fecal occult blood test, sigmoidoscopy, or colonoscopy among adults 50-75y	Fig. 2		Colon screenings				
	63	Mammography use among women 50-74y	Fig. 2		Mammogram screenings				
	64	Men >=65y up to date on core set of preventive services: Flu shot past year, PPV shot ever, Colorectal cancer screening			Older men w/ preventative care				
	65	Women >=65y up to date on core set of preventive services: Flu shot past year, PPV shot ever, Colorectal cancer screening, and Mammogram past 2 years			Older women w/ preventative care				
	66	Taking medicine for high blood pressure control among adults with high blood pressure			High blood pressure controlled				
	67	Visits to dentist or dental clinic among adults	Fig. 2	Fig. 3	Dentist visits				
	68	Visits to doctor for routine checkup within the past year among adults	Fig. 2	Fig. 3	Routine medical checkups				
Negative Adult Health Indicators									
	69	Arthritis among adults			Arhritis	CDC (25)	(See Metric name)	(not needed)	Obtained at tract level.
	70	Binge drinking among adults	Fig. 2	Fig. 3	Binge drinking				
	71	Current asthma among adults	Fig. 2		Asthma				
	72	Current lack of health insurance among adults 18-64y	Fig. 2	Fig. 3	Lacking health insurance				
	73	Current smoking among adults	Fig. 2	Fig. 3	Smoking				
	74	Fair or poor self-rated health status among adults			Self-rated fair or poor health				
	75	Mental health not good for >=14 days among adults	Fig. 2	Fig. 3	Poor recent mental health				
	76	No leisure-time physical activity among adults	Fig. 2	Fig. 3	Lacking leisure physical activity				
	77	Obesity among adults	Fig. 2		Obesity				
	78	Physical health not good for >=14 days among adults			Poor recent physical health				
	79	Sleeping less than 7 hours among adults			Sleeping less than 7 hours				
	80	High blood pressure among adults	Fig. 2	Fig. 3	High blood pressure				
	81	High cholesterol among adults screened in the past 5y	Fig. 2		High cholesterol				

Table S3 continued on next page.

Table S3, continued (page 3 of 3)

Theme	N	Metric	Shown in:		Our Raw Name	Source	Dataset Name	Pre-processing	Tract-aggregation
Adult Disease Outcomes									
	82	LIFEEXPPCT	Fig. 2	Fig. 3	Low life expectancy	EPA (21)	(See Raw Name)	(not needed)	Obtained at tract level.
	83	Depression among adults	Fig. 2	Fig. 3	Depression	CDC (25)	(See Metric name)	(not needed)	Obtained at tract level.
	84	Chronic kidney disease among adults			Kidney disease				
	85	Chronic obstructive pulmonary disease among adults			COPD				
	86	Coronary heart disease among adults	Fig. 2	Fig. 3	Heart disease				
	87	Diagnosed diabetes among adults	Fig. 2	Fig. 3	Diabetes				
	88	Stroke among adults	Fig. 2	Fig. 3	Stroke				
	89	Cancer (excluding skin cancer) among adults	Fig. 2	Fig. 3	Cancer				
	90	Teeth lost 18+			Adult teeth lost				
Adult Disability Prevalence									
	91	Any disability age 18+	Fig. 2		Any disability	CDC (25)	(See Metric name)	(not needed)	Obtained at tract level.
	92	Cognitive disability among adults	Fig. 2	Fig. 3	Cognitive disability				
	93	Hearing disability among adults	Fig. 2	Fig. 3	Hearing disability				
	94	Independent living disability among adults	Fig. 2	Fig. 3	Independent living disability				
	95	Mobility disability among adults	Fig. 2	Fig. 3	Mobility disability				
	96	Self-care disability among adults	Fig. 2	Fig. 3	Self-care disability				
	97	Vision disability among adults	Fig. 2	Fig. 3	Vision disability				
Age demographics									
	98	Median age	Fig. 2		Median age	US Census ACS (26)	MED_AGE	(not needed)	Obtained at tract level.
	99	% Population under age 5	Fig. 2		Percent under age 5		AGE_0_P		
	100	% Population age 5-17	Fig. 2		Percent age 5-17		AGE_5_P		
	101	% Population age 18-34	Fig. 2		Percent age 18-34		AGE_18_P		
	102	% Population age 35-64	Fig. 2		Percent age 35-64		AGE_35_P		
	103	% Population aged 65-74	Fig. 2		Percent aged 65-74		AGE_65_P		
	104	% Population aged 75+	Fig. 2		Percent aged 75+		AGE_75_P		

b. Environment and health data pre-processing

For each of the 104 environment or health metrics considered by this study, source data were either obtained at the census tract level, or were aggregated to the census tract level as means (e.g., tree canopy percent cover) or totals expressed as both area-density and per capita-density metrics (e.g., public transit stops per tract km^2 and per 1000 tract residents).

Simple calculations are noted above in Table S3 and this workflow is depicted in Figure S1.

More involved pre-processing details for relevant datasets are noted in the following sub-sections.

i. Street Tree and Park Tree Inventory data

Within the bounds of the City of Portland, public tree inventories have been in progress for over a decade. The inventory database, as of 2021, for most street trees and public park trees of the city, was used to provide one perspective on the extent of tree canopy and arboreal nature in the city. Data were obtained from the City of Portland (19). A total of 36,924 individual trees were used from the combined inventories.

For attributes Tree Diameter at Breast Height (DBH), Tree Height, and *Tree Crown Width*, the individual trees' records were aggregated to the tract scale by taking the mean of all tree points located inside each tract.

For the attribute *Inventoried Tree Cover Percent (Buffer percent)*, each tree point was first surrounded by a 5m-radius circle, as an estimate of the generic average tree canopy extent per tree point. The total area covered by all these estimated tree crowns in each tract was divided by the total area of the tract, to yield the estimated inventoried tree cover percent metric. As private-property and riparian/greenspace/natural area trees were not part of the inventories, these cover estimates are expected to under-estimate the actual tree canopy coverage, but were still used as an available, systematic means to provide an estimate of the relative public municipal investment in such visible public trees as street and park trees, and as such investment may vary from tract to tract.

ii. Parks and Greenspace, Rural Reserves, Urban Reserves

GIS polygon datasets downloaded from METRO RLIS (14) were:

- Outdoor_Recreation_and_Conservation_Areas_(ORCA) [ORCA_Areas]
- ORCA_Sites
- Urban_and_rural_reserves

Parks and Greenspace:

The ORCA_Sites and ORCA_Areas datasets were very similar but not identical.

The ORCA_Areas data appeared to consist of polygons equivalent to tax parcels. There were several useful categorization attribute fields in the ORCA_Areas dataset: CONSERVATI had values High, Medium, Low; STATUS had values Open, Open_Fee, Open_Restricted, Planned, Unknown; OWNLEV1 had values Bureau of Indian Affairs, Federal, Local, Non-Profits, Private, Special District, State, Unknown; UNITTYPE had values Cemetery, Golf Course, Home Owners Association, Natural Area, Other, Park, School Land.

The ORCA_Sites data appeared to mainly have dissolved contiguous parcels, such as contained in the ORCA_Areas data, into one site. All 14,910 ORCA_Areas polygons intersected at least one ORCA_Sites polygon; however, of the 6,410 ORCA_Sites, 253 did not contain any ORCA_Areas polygon, and 154 did not intersect any ORCA_Areas polygon. The useful categorization attribute of the ORCA_Sites dataset was the attribute field TYPE, with available values: Cemetery, Golf Course, Home Owner Association, Other, Park and/or Natural Area, School.

The ORCA_Areas and ORCA_Sites datasets were combined using the ArcGIS Pro Analysis tool "Union". A new attribute field TYPE_UNION was added to combine the ORCA_Areas UNITTYPE and ORCA_Sites TYPE fields according to the following sequential method:

- if either attribute included 'school', the TYPE_UNION was set to School;
- if either attribute included 'Home Owners Association', the TYPE_UNION was set to HOA;

- if either attribute included ‘Cemetery’, the TYPE_UNION was set to Cemetery;
- if either attribute included ‘Golf’, the TYPE_UNION was set to Golf;
- if the ORCA_Areas UNITTYPE was ‘Park’ or ‘Natural Area’ or the ORCA_Sites TYPE was ‘Park and/or Natural Area’, the TYPE_UNION was set to Park.
- The remaining (n=1148) polygons all had ORCA_Sites TYPE “Other” and ORCA_Areas UNITTYPE “Other” or blank.
  - If the ORCA_Sites NAME included the words “Reservoir” or “Dam” or “Water” (Water Tank or Water Quality Facility) or “Maintenance” the TYPE_UNION was set to “Non-rec” (23 polygons).
  - If the ORCA_Areas OWNER included the word “School” the TYPE_UNION was set to “School” (3 polygons).
  - Polygons of this ‘other’ type that were at least 100,000 sq. ft. polygon area (about 0.9 hectares) were inspected individually for the presence of housing development or other key identifying features as seen in the ArcGIS ESRI “World Topographic Map” basemap (version accessed: September 2024):
    - six polygons that appeared to now be housing developments were given TYPE_UNION set to HOA;
    - four polygons that appeared to be water tank properties (one not >100K sq.ft. but adjacent, of ‘Other’ category, and part of the same apparent tank building as larger adjacent such polygon) were set to “Non-rec”.
    - The remainder of these larger polygons (area at least 100,000 sq. ft.) all appeared to be various municipal and development open spaces; these polygons, and all remaining smaller polygons with ORCA_Sites TYPE “Other” and no assigned TYPE_UNION value were given TYPE_UNION set to Park.

All ORCA polygons with TYPE_UNION not equal to “Non-rec” were selected, then dissolved on the field TYPE_UNION, creating a final dissolved layer not including the “Non-rec” properties; in other words, the final analysis retained all ORCA polygons of TYPE_UNION: School, HOA, Cemetery, Golf, Park, or Other (mostly open space).

Urban and rural reserves:

According to the regional planning jurisdiction METRO: “Urban reserves are lands suitable for accommodating urban development over the 50 years after their designation. Rural reserves are lands that are high value working farms and forests or have important natural features like rivers, wetlands, buttes and floodplains. These areas will be protected from urbanization for 50 years after their designation.” (27)

Combining greenspace:

The dissolved ORCA layer and the downloaded Urban_and_rural_reseves layer were each cut at census tract boundaries using the ArcGIS Pro Analysis tool “Pairwise Intersect”, then polygon areas freshly calculated using the ArcGIS feature attribute table field “Calculate Geometry” function “Area” with units square meters and with projection NAD_1983_UTM_Zone_10N and saved in the new field Area_km2. To associate the ORCA areas to the census tract polygons, the ArcGIS feature modification option “Add Spatial Join” was used on the census tracts, directing the tool to use the ORCA polygons contained by each tract, and using the tool’s ‘Output Fields’ options to return the sum of the field Area_km2 for each tract. This procedure was repeated for the Reserve polygons, but first separately selecting only the rural reserves, then repeating the procedure after selecting only the urban reserves, to tabulate each separately to the tract level.

The total areas per tract of each of these three categories were then saved as permanent attributes in the census tract shapefile as ORCAkm2, RReskm2, and UReskm2. Area proportion (areal density) of each of these per census tract was calculated by dividing it by the census tract area in square km, to produce tract attributes ORCAdens, RResDens, UResDens. Land area in each of these three categories per population (per 1000 residents) was calculated for each census tract as the ORCAkm2, RReskm2, and

UReskm2 values, times 1000, divided by the census tract Census 2010 population, to produce tract attributes ORCApp, RResPP, UResPP.

iii. Tree canopy greenness and chlorophyl density

High spatiotemporal resolution multi-spectral imagery (Planetscope, 3m-resolution, R-G-B-N-RE, PSOrtho5b) was used to assess responses of urban trees around the time of a record-breaking heat wave event in the Pacific Northwest in summer 2021. Orthorectified and surface reflectance-corrected R-G-B-NIR-RedEdge from two imagery dates, immediately before the heatwave (June 20, 2021) and shortly after (July 29, 2021), were obtained from PlanetLabs (20) and mosaiced across the city. This, and further spatial processing as noted below, was conducted using QGIS software (QGIS.org, Version 3.14-PI). Reflectance values were used to map urban greenery using the normalized-difference vegetation index (NDVI) calculated each pixel of each mosaiced satellite image:

$$NDVI = (NIR - Red) / (NIR + Red)$$

Healthier, greener trees have NDVI values closer to 1; brown or bare surfaces have NDVI closer to zero.

A mask to capture identifiable tree crown greenery was derived by using the known point-locations of 36,924 trees mapped by the recent city-wide street tree and park tree inventory programs of the City of Portland (19), and buffering each tree point location with a 5m circle. The intersection of each NDVI raster and the tree canopy mask was used to select the Planetscope imagery pixels intersecting each tree crown circle (n = 5 on average) and then calculate the mean NDVI value of each such tree crown for each imagery date. Finally, mean per-tree NDVI values were calculated for each census tract.

The above procedure was then repeated using the alternative canopy metric, the *normalized-difference in red edge (NDRE)* calculated each pixel of each mosaiced satellite image:

$$NDRE = (NIR – RedEdge) / (NIR + RedEdge)$$

iv. Transportation Noise

Although not a precise, location-specific noise model, the simplified model of the National Transportation Noise Map (23) was considered sufficient for exploring potential relative differences in noise burden at the coarse scale of census tracts.

The ArcGIS Pro Spatial Analyst tool “Zonal Statistics as Table” was used to calculate the statistics (mean, standard deviation, median, pixel count) of the transportation noise map pixel values corresponding to each of the census tract polygons, using the tract FIPS number as each polygon’s unique zonal identifier, a cell size of 10m, snapping cells to the input noise level dataset, and projection NAD_1983_UTM_Zone_10N.

Noise level was summarized to the tract level as the mean, standard deviation, median, and maximum of the values of cells the centers of which fell within the rasterized tract polygon boundary, from the Zonal Statistics tool output.

v. Building footprints, area, and density

Using the METRO Building Footprints dataset (14), the footprints polygons were first combined with the census tract polygons using the ArcGIS Pro “Union” tool, so buildings footprints spanning tract boundaries would be cut into separate footprint portions along the boundary.

Fresh building footprint areas (BldAm2) were calculated in square meters by using the “Calculate Geometry” tool in the feature attribute table with units m and with projection NAD_1983_UTM_Zone_10N, to accurately calculate any newly split, partial footprint areas. Values of zero in the YEAR_BUILT attribute were reclassified to <Null>.

Total building area per census tract in sq. m (BldAm2) was calculated using the ArcGIS Statistics Toolset tool “Summarize Within”; in the same calculation, mean of YEAR_BUILT was also calculated for each tract; projection used was NAD_1983_UTM_Zone_10N. A new attribute was calculated BldAsum = sum(BldAm2)/1,000,000 (to convert to square km units). Building Area Density (BldAdens) was calculated as the BldAsum of each census tract, divided by the total census tract area (in units sq km / sq km). Building Area per population (BldApp) was calculated as the BldAsum of each census tract, times 1000 (per 1000 residents), divided by the census tract Census 2010 population.

vi. Estimated air temperatures

Modeled raster maps of urban air temperatures were obtained from the public archiving of the "Heat Watch Portland Metro Region" analysis (22), which conducted a field campaign to survey air temperature traverses throughout the city on 22-July-2023 at 6-7am, 3-4pm, and 7-8pm. Those field data were combined with Sentinel-2 remote sensing data in a statistical model to estimate metro-wide air temperature maps for those three times of the study day, which was a hot summer day in the local climate. The metadata with this dataset refers the reader to the additional references (28, 29).

vii. Transit Stations

GIS transit stop datasets downloaded from METRO RLIS (14) were:

- TriMet bus system stops
- TriMet Park & Ride lots
- Light rail stations
- Rail transit stations
- TriMet transit centers

It was determined that the "Light rail stations" and "Rail transit stations" were duplicate points, so only one of the two sets of points was used. The remaining four types of points (bus stops, park & ride lots, rail stations, and transit centers) were merged into a single points shapefile using the ArcGIS Pro tool "Merge".

Some points from the four different sources were spatially identical or nearly identical in location. It was also decided to count two bus stops on opposite sides of the same street as a single 'station'. Most such paired bus stops were about 35 m or less apart, but some were further apart when off-set by perhaps half a block. These two types of duplicate points were removed using the ArcGIS Pro 3.1.3 tool "Delete Identical" with "XY Tolerance" of 60 meters, i.e., for every two points located less than 60 m apart, the tool removed one of the points. The smaller dimension of most city blocks is greater than 60 meters, so bus stops and other transit points located a block or more apart were thus retained as separate transit access points.

To count the remaining transit stops within an easily accessible distance from each census tract, the ArcGIS feature modification option "Add Spatial Join" was used on the census tracts, using "Match Option" set to "Within a distance" and "Search Radius" of 200 meters. The resulting "Join_Count" field was then retained in association with each census tract as the count of contained and nearby transit stops. The non-zero search radius was used because both transit points and census tract boundaries often fell along major roadways; using a zero search distance, or counting points only inside each census tract, would effectively randomize the inclusion or exclusion of transit stops that might have been reduced to one side of the road or the other by the previous "Delete Identical" trimming step. The 200m search radius was sufficient to include approximately one to three city blocks' distance outside each census tract boundary (depending on block size and orientation), to be inclusive of transit stops or stations functionally very close and accessible to each tract but perhaps not geocoded or trimmed to remain within the tract. This method does mean that transit stops within 200 m of adjacent census tracts' boundary are likely counted twice, once for each neighboring tract, but this is by design, not a mistake, as the intent is to identify the availability of public transit to the residents in each tract, regardless of those stops' availability to residents of other tracts.

Transit point density was calculated as this final count of transit stops in/near each census tract divided by the census tract area in square km. Tract areas in square km were calculated using the ArcGIS feature attribute table field "Calculate Geometry" function "Area" with units km and with projection NAD_1983_UTM_Zone_10N. Transit points per population (per 1000 residents) was calculated as the final count of transit stops in/near each census tract, times 1000, divided by the census tract Census 2010 population.

viii. Transit Routes

GIS transit route datasets downloaded from METRO RLIS (14) were:

- Light_rail
- TriMet_Bus_System_(routes)
- Bike_Routes
- Sidewalks
- Trails

In the Light Rail data: The GIS feature attribute STATUS was used to identify active light rail lines. A new attribute field RAIL_PCT was added to a copy of the dataset and scored as 200 for STATUS “Existing” and 0 for STATUS “Planned”.

In the Bus Routes data: The GIS feature attribute FREQUENT was used to identify core and frequent versus less active bus lines. A new attribute field SCORE_BUS was added to a copy of the dataset and scored as 200 for FREQUENT “TRUE” and 100 for FREQUENT “False”.

In the Bike Routes data: There appeared three overlapping but not entirely identical bike route designation systems: BIKETYPE, BIKETHERE, and SYMB_CAT. A new attribute field SCORE_BK was added to a copy of the dataset and filled as follows. Line segments with all three of these designations blank were assigned SCORE_BK = 0. All but two line segments with BIKETHERE = “CA” corresponded to SYMB_CAT = “Bike with caution” (the other two were blank in SYMB_CAT); these were types of routes that analysts of this study would not feel safe biking, e.g., due to narrow and steep busy roads, highway conditions, or arterials without notable bike lanes or traffic slowing features. Consequently, all line segments with BIKETHERE = “CA” were assigned SCORE_BK = 0. The remainder of line segments with SYMB_CAT = “Bike with caution” had BIKETHERE = “HT” (high traffic) and were assigned SCORE_BK = 30. The remainder of line segments with BIKETHERE = “HT” had SYMB_CAT = “Dedicated bike lane - bike with caution” and were assigned SCORE_BK = 60. The remainder of the SYMB_CAT designations (“Bike friendly street”, “Dedicated bike lane”, “Established bikeway”, “Helpful connection”, and “Local trail”) were assigned SCORE_BK = 100.

In the Sidewalks data: Availability of sidewalks was identified by the attributed table fields LPCT and RPCT. A new attribute field PCT_SW was added to a copy of the dataset, and calculated as (PCT_SW) = [(LPCT + RPCT) / 2]. Availability of sidewalks was conceptually validated by the field SYMB_CAT: Line segments with LPCT = 100 and RPCT = 100 (suggesting 100 percent coverage of both left and right street margins by sidewalks) appeared in the dataset with SYMB_CAT = “Full Sidewalk Both Sides”. Almost all line segments with SYMB_CAT = “Partial Sidewalk” had a PCT_SW of about 50, typically due to sidewalk on one side but not the other (median 50, mean 53, st.dev. 15). Almost all line segments with SYMB_CAT = “Missing Sidewalk” had PCT_SW close to zero (median 0, mean 2, st.dev. 7).

In the Trails data: A new attribute field SCORE_TR was added to a copy of the dataset and scored according to the following rules. SCORE_TR = 0 for line segments with field STATUS of “Conceptual”, “Decommissioned”, “Planned”, or “Restricted_Private” or with SYMB_CAT of “Planned trail” or “Conceptual trail”. SCORE_TR = 50 for line segments with field STATUS of “Open_Fee”, mostly regional (METRO) parks, state parks, and federal national forests. SCORE_TR = 100 for line segments with field STATUS of “Open”. The remaining 42 of the 26,311 line segments in the dataset had STATUS of “Under Construction” (n=40) or “Unknown” (n=2) and SYMB_CAT blank; these were coded SCORE_TR = 0.

These five sets of line features were then combined using the Data Management tool “Merge” and then cut at census tract boundaries by the Analysis tool “Pairwise Intersect”. All line segment lengths in the merged dataset were then freshly calculated using the ArcGIS feature attribute table field “Calculate Geometry” function “Length” with units meters and with projection NAD_1983_UTM_Zone_10N.

A length-weighted score for each transit line segment was then calculated in a new field “TR_LN_SCR” as the score from the line segment’s transit type (as developed above: RAIL_PCT, SCORE_BUS, SCORE_BK, PCT_SW, or SCORE_TR) times the line segment length, divided by 100 (to convert the percent-like scores to fractions).

To associate these transit route scores with each census tract, the ArcGIS feature modification option “Add Spatial Join” was used on the census tracts, using “Match Option” set to “Within a distance” and “Search Radius” of 30 meters. This non-zero search radius was used intentionally, so as to count a transit line directed coincidentally along the boundary between census tracts (such as bus routes along major roads, which also split census tracts) as equally accessible to both of the adjacent census tracts, and to

mitigate the effects of any small route/tract spatial misalignments. The tool was directed to return the sum of the TR_LN_SCR values captured by this spatial join and search radius for each census tract.

The per-tract sum of transit route segment scores was then divided by 1000 (to convert meter-like units to km-like units), and saved at the tract level as new field “TransRteScore”. Transit route density was calculated as TransRteScore divided by the census tract area in square km. Transit route availability per population (per 1000 residents) was calculated as TransRteScore, times 1000, divided by the census tract Census 2010 population.

ix. Community Buildings

GIS datasets downloaded from METRO RLIS (14) were:

- Community_Centers
- Hospitals
- Libraries
- Fire Stations
- City Halls
- Schools
- School Sites (polygons)

Types of facilities within the METRO Schools points dataset were distinguished by the LEVEL_NAME attribute field. Points with LEVEL_NAME = “Administration,” indicating administrative and not school buildings, were excluded from further analysis.

There were eight polygons in the School Sites dataset that did not overlap with points in the Schools dataset, were not designated in their SITE_TYPE field as “Admin” (administrative building), “Dev” (district property available for school development), or “Vac” (vacant), and appeared from their feature attributes to be actual schools; these eight polygons were converted to point centroids for inclusion in the combined point data assessment.

The seven sets of points representing community buildings were merged into a single points shapefile using the ArcGIS Pro tool “Merge”. To count the built amenities within an easily accessible distance from each census tract, the ArcGIS feature modification option “Add Spatial Join” was used on the census tracts, using “Match Option” set to “Within a distance” and “Search Radius” of 1000 meters. The resulting “Join_Count” field was then retained in association with each census tract as the count of contained and nearby built amenities.

Community building point density (ComBldgPtsDens) was calculated as this count of community resources in/near each census tract divided by the census tract area in square km. Community buildings per population (per 1000 residents) was calculated as the count, times 1000, divided by the census tract Census 2010 population (ComBldgPtsPP).

c. Neighborhood Index and HOLC relations to environment and health data

We explored the contrasts between and relationships among the typologies, the HOLC-zone areas, and a wide range of environmental and human health indicator variables. We quantified significant relations in two ways.

First, we calculated Spearman’s correlation coefficients between each continuous-valued environmental and or health metric and our continuous-valued indices and HOLC scores. We assessed the correlation coefficients both in terms of correlation magnitude and significance (see manuscript Figure 2), and generally used a very low threshold for significance to account for the statistical issue of multiple hypothesis testing.

Second, we extracted the six subsets of census tracts most characteristic of the key populations identified by each of the four indices (tracts scored with exclusivity, disadvantage, gentrification, or displacement-hosting index values > 1) and by high or low HOLC scores (> 0.75 and < -0.75, respectively), and calculated the mean value of each environmental or health metric among each of those six subsets of tracts, as well as the mean of all tracts in the historical HOLC-zoned area, and the overall mean of all tracts (see manuscript Figure 3). We then the assessed the ratios and absolute differences between pairs of these means, and focused on differences with effect sizes > 0.8. Effect size was as by standard mean difference, calculated conservatively by the difference in the two typologies’ means for the metric of

interest divided by the all-tract standard deviation of per-tract values of that metric. We illustrated the relative magnitudes of these means for selected variables (see manuscript Figure 3), after transforming the set of means to within-set z-scores to normalize the scales of the diverse variables.

SUPPORTING INFORMATION: RESULTS

D. Results of neighborhood indexes' derivations

Table S4. ACS attributes' PCA loadings on each neighborhood typology index

The PCA eigenvector for each index shows the loadings of each attribute's relative contribution to the index. Attribute values from 2010 and 2020 were both separately used as inputs to the exclusivity PCA to derive the Persistent Exclusive Index, to seek an index representing high values in both years. Differences between attribute values in 2010 and 2020 were used as the inputs to the gentrification PCA and the displacement PCA, to derive those indices as characterized by magnitude and direction of change over time.

**Persistent Exclusivity Index (inverse used as Persistent Disadvantage Index)**		**Gentrification Index**		**Displacement-hosting Index**	
PCA input attribute	PCA eigenvector (loadings)	PCA input attribute	PCA eigenvector (loadings)	PCA input attribute	PCA eigenvector (loadings)
% individuals with at least bachelor's degree in 2010, 2020	0.39, 0.38	Increase in % individuals with at least bachelor's degree (2020-2010)	0.61	Increase in % individuals without bachelor's degree (2020-2010)	0.48
% individuals identifying as white-only in 2010, 2020	0.29, 0.29	Increase in % individuals identifying as white-only (2020-2010)	0.34	Increase in % individuals not identifying as white-only (2020-2010)	0.37
% households above median income in 2010, 2020	0.34, 0.36	Increase in % households above median income (2020-2010)	0.61	Increase in % households below median income (2020-2010)	0.50
% buildings built before 2010	-0.06	% buildings built 2010 through 2019	0.35	% increase median rent value (2020-2010)/2010	0.18
Median home value in 2010, 2020	0.38, 0.38	Increase in % multi-family housing (2020-2010)	0.13	% increase median home value (2020-2010)/2010	0.39
-		-		Increase in % renter occupied households (2020-2010)	0.26
-		-		Increase in % households speaking limited English (2020-2010)	0.36

a. Persistently Exclusive Areas PCA results

Persistent exclusivity was indicated in roughly equal measure by high home values, household incomes, bachelor’s degree attainment, and fraction of tract population identifying as white-only, in both 2010 and 2020 (Table S4, above; Figure S2, below).

The opposite trends in these attributes indicated persistently disadvantaged areas.

b. Gentrified Areas PCA results

Recent gentrification was indicated by a transition toward more socioeconomically advantaged characteristics between 2010 and 2020, dominantly due to increased household incomes and bachelor’s degree-attainment, and secondarily from increased fractions of the population identifying as white-only and of homes built since 2010 (Table S4, above; Figure S3, below). Surprisingly, an increase in the proportion of multifamily housing between 2010 and 2020 was only very weakly related to the typology of gentrification in the study region.

c. Displacement-hosting Areas PCA results

The transition of some census tracts toward more socioeconomically disadvantaged characteristics between 2010 and 2020 reflected new geographic aggregations of typically marginalized individuals, whether newcomers settling in these areas, or existing area residents voluntarily or involuntarily displaced from other exclusive, gentrifying, or disadvantaged neighborhoods. These increasingly displacement-hosting areas captured signals of increasing disadvantage from, primarily, decreased household incomes and bachelor’s degree-attainment at the same time as increased median home values and, secondarily, from increased proportions of households speaking limited English, individuals not identifying as white-only, and housing units occupied by renters, at the same time as increased median rent values (Table S4, above; Figure S4, below). The economic pinch of these displacement-hosting areas, exhibiting decreased household incomes and increased proportions of renters amid simultaneously increasing home values and increasing rents was particularly notable.

Principal Components: on Correlations

Correlations

	bach_yes_pct_10_z	bach_yes_pct_20_z	inc_over_med_pct_10_z	inc_over_med_pct_20_z	re_nh_caw_pct_10_z	re_nh_caw_pct_20_z	medval_10_z	medval_20_z	built_2010pre_pct_z
bach_yes_pct_10_z	1.0000	0.9135	0.6080	0.6432	0.4583	0.4051	0.7756	0.7453	-0.1697
bach_yes_pct_20_z	0.9135	1.0000	0.5134	0.6717	0.4088	0.4274	0.6967	0.7580	-0.2452
inc_over_med_pct_10_z	0.6080	0.5134	1.0000	0.8261	0.4663	0.4138	0.6291	0.5606	0.0290
inc_over_med_pct_20_z	0.6432	0.6717	0.8261	1.0000	0.4560	0.4746	0.5948	0.6538	-0.1100
re_nh_caw_pct_10_z	0.4583	0.4088	0.4663	0.4560	1.0000	0.7620	0.4929	0.4596	0.0207
re_nh_caw_pct_20_z	0.4051	0.4274	0.4138	0.4746	0.7620	1.0000	0.4505	0.4875	0.0220
medval_10_z	0.7756	0.6967	0.6291	0.5948	0.4929	0.4505	1.0000	0.8481	-0.1137
medval_20_z	0.7453	0.7580	0.5606	0.6538	0.4596	0.4875	0.8481	1.0000	-0.0682
built_2010pre_pct_z	-0.1697	-0.2452	0.0290	-0.1100	0.0207	0.0220	-0.1137	-0.0682	1.0000

The correlations are estimated by Row-wise method.

Summary Plots

Eigenvalue	20 40 60 80
5.2086	
1.2285	
0.8974	
0.6698	
0.3792	
0.2977	
0.1804	
0.0885	
0.0499	

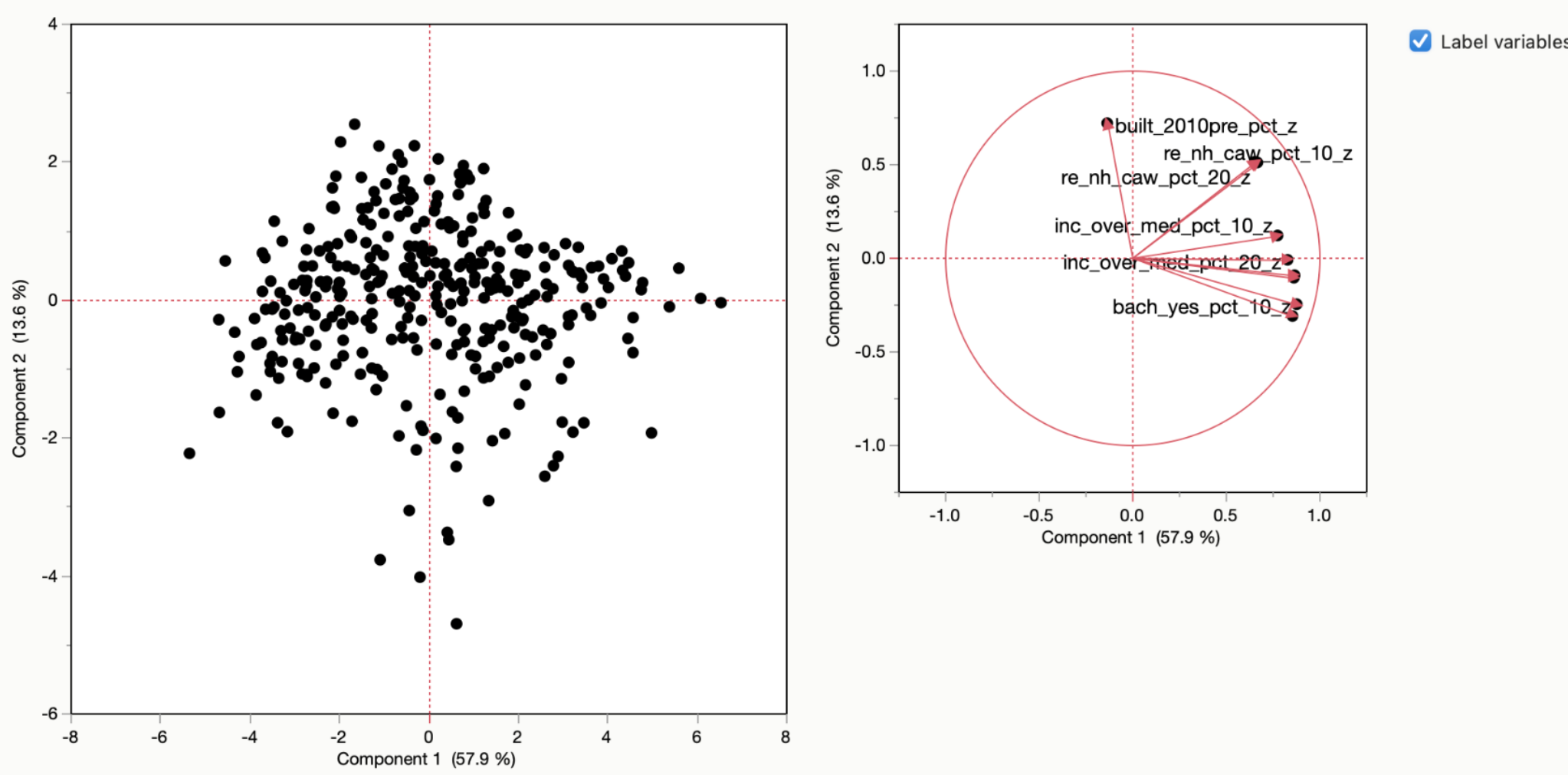

Select component Component 1 Component 2

Eigenvalues

Number	Eigenvalue	Percent	20 40 60 80	Cum Percent
1	5.2086	57.873		57.873
2	1.2285	13.650		71.523
3	0.8974	9.971		81.494
4	0.6698	7.442		88.936
5	0.3792	4.214		93.150
6	0.2977	3.308		96.457
7	0.1804	2.005		98.462
8	0.0885	0.984		99.446
9	0.0499	0.554		100.000

Eigenvectors

	Prin1	Prin2	Prin3	Prin4	Prin5	Prin6	Prin7	Prin8	Prin9
bach_yes_pct_10_z	0.38579	-0.22473	0.09256	0.22684	0.36621	-0.35917	-0.23634	-0.07528	-0.64498
bach_yes_pct_20_z	0.37590	-0.28170	0.00599	0.22592	0.53701	0.05304	0.04798	0.01650	0.65917
inc_over_med_pct_10_z	0.34089	0.10650	0.31660	-0.58036	-0.15338	-0.24865	-0.31129	-0.44951	0.22602
inc_over_med_pct_20_z	0.36435	-0.01084	0.17485	-0.53328	0.16010	0.34172	0.35765	0.47983	-0.22720
re_nh_caw_pct_10_z	0.29352	0.45798	-0.43473	0.01029	-0.02192	-0.51655	0.49479	-0.01145	0.05245
re_nh_caw_pct_20_z	0.28744	0.46153	-0.46266	0.03543	0.10017	0.46402	-0.51035	-0.00994	-0.05617
medval_10_z	0.37921	-0.09707	0.10822	0.26618	-0.59548	-0.16747	-0.25044	0.54346	0.15328
medval_20_z	0.38074	-0.08515	0.12621	0.32511	-0.35269	0.42344	0.38535	-0.50749	-0.12817
built_2010pre_pct_z	-0.05810	0.64750	0.65567	0.31366	0.19709	0.01022	0.02297	0.09294	0.03100

Figure S2. Persistent Exclusivity Index PCA derivation results

The subsections of the above figure are the following:

- *Correlations*: Correlations among the z-transformed index PCA input variables.
- *Summary Plots*: Illustrating data scatter for PC1 vs PC2 and variable loadings on PC1 and PC2.
- *Eigenvalues*: Portion of the total variance of the multidimensional index input space explained by each principal component and the cumulative percent variance explained.
- *Eigenvectors*: Loading table showing relative weights of index input variables' contributions to each principal component. PC1 (Prin1) was used as the Index.

Principal Components: on Correlations

Correlations

	bach_yes_diff_z	inc_over_med_diff_z	re_nh_caw_diff_z	built_2010plus_diff_z	ht_attached_diff_z
bach_yes_diff_z	1.0000	0.5083	0.2541	0.1661	0.0567
inc_over_med_diff_z	0.5083	1.0000	0.1811	0.2339	0.0145
re_nh_caw_diff_z	0.2541	0.1811	1.0000	-0.0123	-0.0367
built_2010plus_diff_z	0.1661	0.2339	-0.0123	1.0000	0.1886
ht_attached_diff_z	0.0567	0.0145	-0.0367	0.1886	1.0000

The correlations are estimated by Row-wise method.

Summary Plots

Eigenvalue
1.7525
1.1687
0.8696
0.7324
0.4768

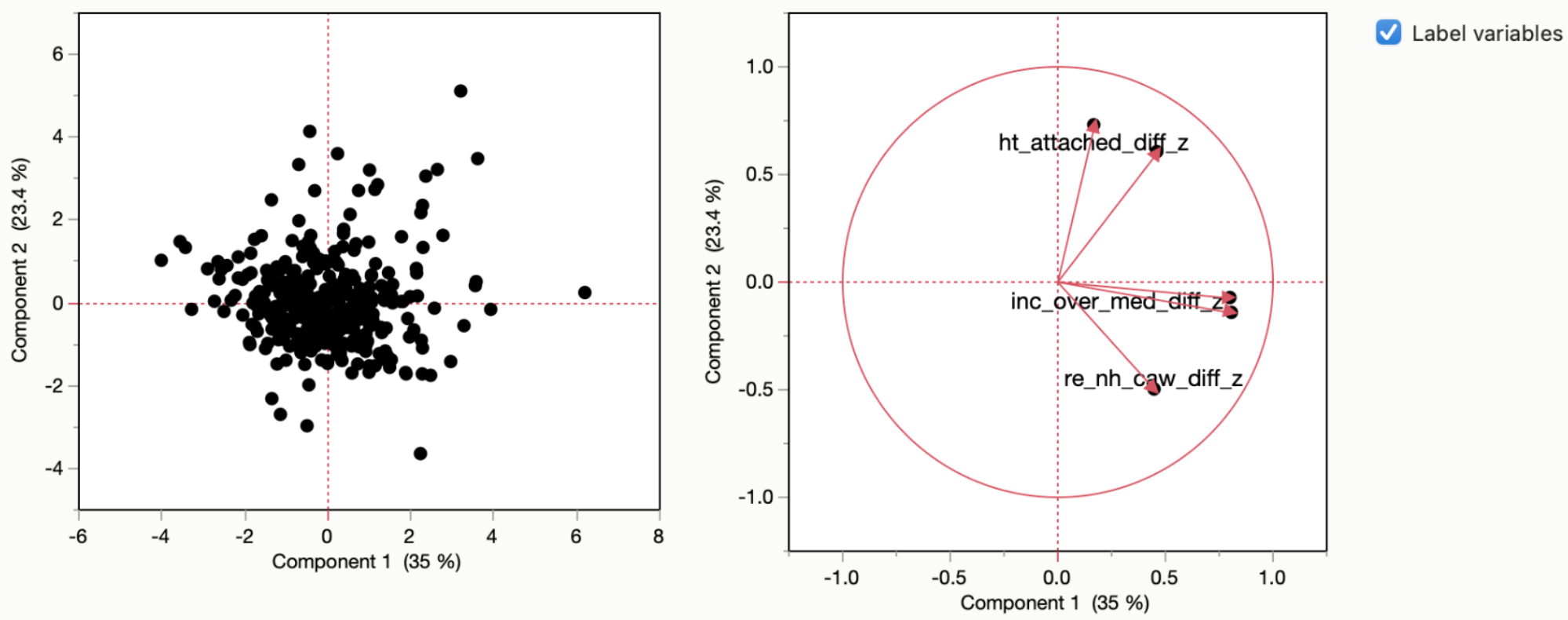

Select component Component 1 Component 2

Eigenvalues

Number	Eigenvalue	Percent	Cum Percent
1	1.7525	35.050	35.050
2	1.1687	23.375	58.425
3	0.8696	17.391	75.816
4	0.7324	14.648	90.463
5	0.4768	9.537	100.000

Eigenvectors

	Prin1	Prin2	Prin3	Prin4	Prin5
bach_yes_diff_z	0.61253	-0.13370	-0.04597	-0.35029	-0.69435
inc_over_med_diff_z	0.60734	-0.06992	-0.29345	-0.24574	0.69264
re_nh_caw_diff_z	0.34093	-0.46324	0.65061	0.48524	0.10209
built_2010plus_diff_z	0.35074	0.55713	-0.27992	0.68728	-0.12606
ht_attached_diff_z	0.12920	0.67250	0.64041	-0.33031	0.10872

Figure S3. Recent Gentrification Index PCA derivation results

The subsections of the above figure are the following:

- *Correlations*: Correlations among the z-transformed index PCA input variables.
- *Summary Plots*: Illustrating data scatter for PC1 vs PC2 and variable loadings on PC1 and PC2.
- *Eigenvalues*: Portion of the total variance of the multidimensional index input space explained by each principal component and the cumulative percent variance explained.
- *Eigenvectors*: Loading table showing relative weights of index input variables' contributions to each principal component. PC1 (Prin1) was used as the Index.

Principal Components: on Correlations

Correlations

	bach_no_diff_z	rent_occupied_diff_z	poc_diff_z	income_diff_z	language_diff_z	medval_chg_z	rentval_chg_z
bach_no_diff_z	1.0000	0.1120	0.2541	0.5083	0.1570	0.4055	0.0937
rent_occupied_diff_z	0.1120	1.0000	0.0056	0.3039	0.2749	-0.0109	0.1355
poc_diff_z	0.2541	0.0056	1.0000	0.1811	0.4811	0.1775	0.0401
income_diff_z	0.5083	0.3039	0.1811	1.0000	0.1848	0.3844	0.1936
language_diff_z	0.1570	0.2749	0.4811	0.1848	1.0000	0.0965	0.0304
medval_chg_z	0.4055	-0.0109	0.1775	0.3844	0.0965	1.0000	0.0708
rentval_chg_z	0.0937	0.1355	0.0401	0.1936	0.0304	0.0708	1.0000

The correlations are estimated by Row-wise method.

Summary Plots

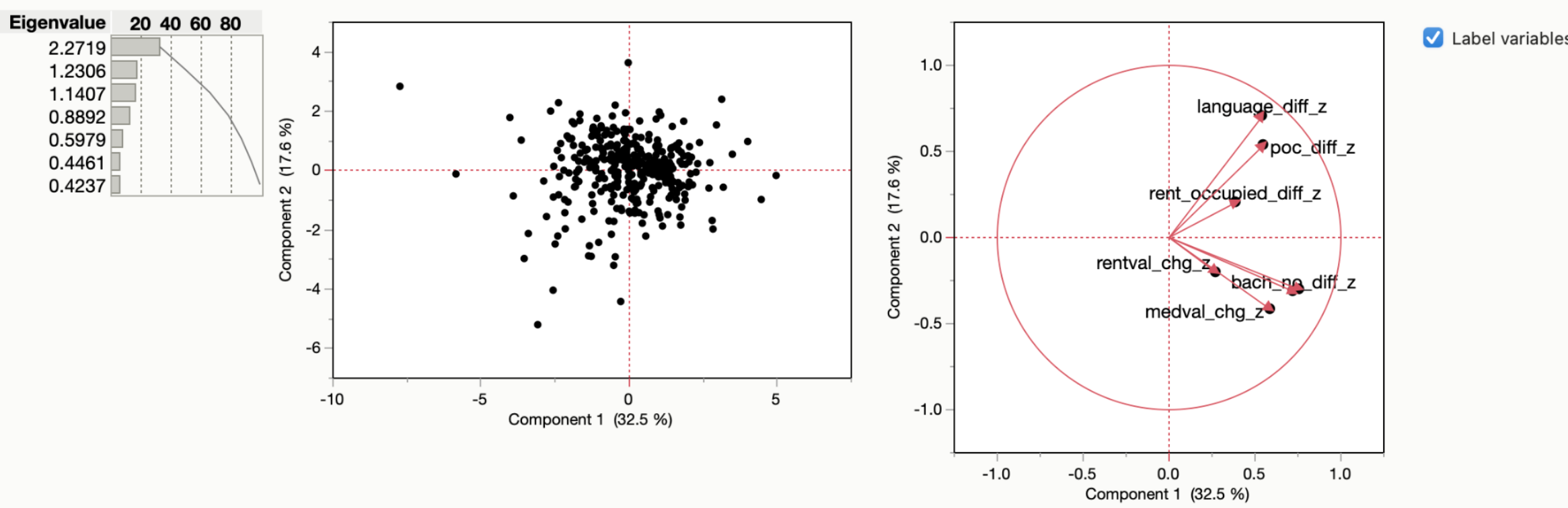

Select component Component 1 Component 2

Eigenvalues

Number	Eigenvalue	Percent	20 40 60 80	Cum Percent
1	2.2719	32.455		32.455
2	1.2306	17.581		50.036
3	1.1407	16.295		66.331
4	0.8892	12.703		79.034
5	0.5979	8.541		87.575
6	0.4461	6.372		93.948
7	0.4237	6.052		100.000

Eigenvectors

	Prin1	Prin2	Prin3	Prin4	Prin5	Prin6	Prin7
bach_no_diff_z	0.47969	-0.28203	-0.17435	-0.11974	-0.53845	0.59326	0.06111
rent_occupied_diff_z	0.25809	0.18338	0.68507	-0.42801	0.15419	0.08549	0.46494
poc_diff_z	0.36538	0.48357	-0.35620	0.30037	-0.17840	-0.32580	0.52687
income_diff_z	0.50371	-0.27227	0.15443	-0.17345	-0.19442	-0.66440	-0.37280
language_diff_z	0.36045	0.63531	0.00460	-0.00060	0.22451	0.27565	-0.58314
medval_chg_z	0.39144	-0.37640	-0.33445	-0.01044	0.75166	0.07071	0.15209
rentval_chg_z	0.18142	-0.18321	0.48752	0.82587	0.03556	0.11037	-0.01860

Figure S4. Recent Displacement-hosting Index PCA derivation results

The subsections of the above figure are the following:

Correlations: Correlations among the z-transformed index PCA input variables.

Summary Plots: Illustrating data scatter for PC1 vs PC2 and variable loadings on PC1 and PC2.

Eigenvalues: Portion of the total variance of the multidimensional index input space explained by each principal component and the cumulative percent variance explained.

Eigenvectors: Loading table showing relative weights of index input variables' contributions to each principal component. PC1 (Prin1) was used as the Index.

E. Relations of HOLC zones and indices

Over the whole metro region, persistent exclusivity or disadvantage was only weakly correlated with displacement-hosting areas and even less correlated with gentrified areas, as befits the largely orthogonal design of the indices (Table S5). Few tracts exhibited overlapping typologies of persistent exclusivity or disadvantage at the same time as either recent gentrification or displacement-hosting (manuscript Figure 1g).

Table S5. Relations between neighborhood typologies and HOLC zones

	HOLC scores in						
	all HOLC-zoned areas	HOLC A & B zones only	HOLC C & D zones only	Gentrification Index	Displacement-hosting Index	Persistent Exclusivity Index	Persistent Disadvantage Index
Spearman correlation coefficient of index (row) with index (column)							
HOLC score	1	1	1	-0.38	0.32	0.48	-0.48
Gentrification Index	-0.38	-0.45	-0.29	1	-0.86	0.01	-0.01
Displacement-hosting Index	0.32	0.51	0.14	-0.86	1	0.18	-0.18
Persistent Exclusivity Index	0.48	0.69	0.09	0.01	0.18	1	-1
Persistent Disadvantage Index	-0.48	-0.69	-0.09	-0.01	-0.18	-1	1
Two-tailed correlation significance							
HOLC score				0.0001	0.0014	0.0000	0.0000
Gentrification Index	0.0001	0.0123	0.0182		0.0000	0.9086	0.9086
Displacement-hosting Index	0.0014	0.0038	0.2558	0.0000		0.0007	0.0007
Persistent Exclusivity Index	0.0000	0.0000	0.4852	0.9086	0.0007		
Persistent Disadvantage Index	0.0000	0.0000	0.4852	0.9086	0.0007		

Comparing the spatial distributions of persistent exclusivity and disadvantage or increased gentrification or displacement-hosting to the historical HOLC zoning, the spectrum of overall HOLC scores derived from the historical HOLC zonation was most useful for predicting persistent contemporary neighborhood exclusivity: tract-level HOLC scores were significantly positively related to the Exclusivity Index (Table S5). However, this relationship was strongly driven by the alignment of high-exclusivity areas with HOLC A and B zones, whereas there was less coherence in the relationship between high-disadvantage areas and HOLC C and D zones (Figure S5).

An overall positive statistical relationship between contemporary census tract persistent exclusivity/disadvantage and historical HOLC zoning exists (top-left of Figure S5). However, this masks that only the relative degree of persistent privilege (exclusivity) among the most highly exclusive tracts is well-predicted by HOLC zoning (see top-right of Figure S5), specifically the distinction between HOLC zones A and B, whereas HOLC C vs D zoning has no predictive power to explain persistent exclusivity or disadvantage today.

In general gentrification has recently been more concentrated in areas with locations more central to historical HOLC D zones (see Figure S5, bottom-left: a fully D-zone tract would have HOLC score -2, whereas a partly D-zone tract would have HOLC score > -2), at least for those gentrifying areas within the historically zoned urban core. Of recent displacement-hosting areas in the urban core, they tend to the opposite, with more recent decline in relative socioeconomic and racial privilege in HOLC A areas (Figure S5, bottom-right).

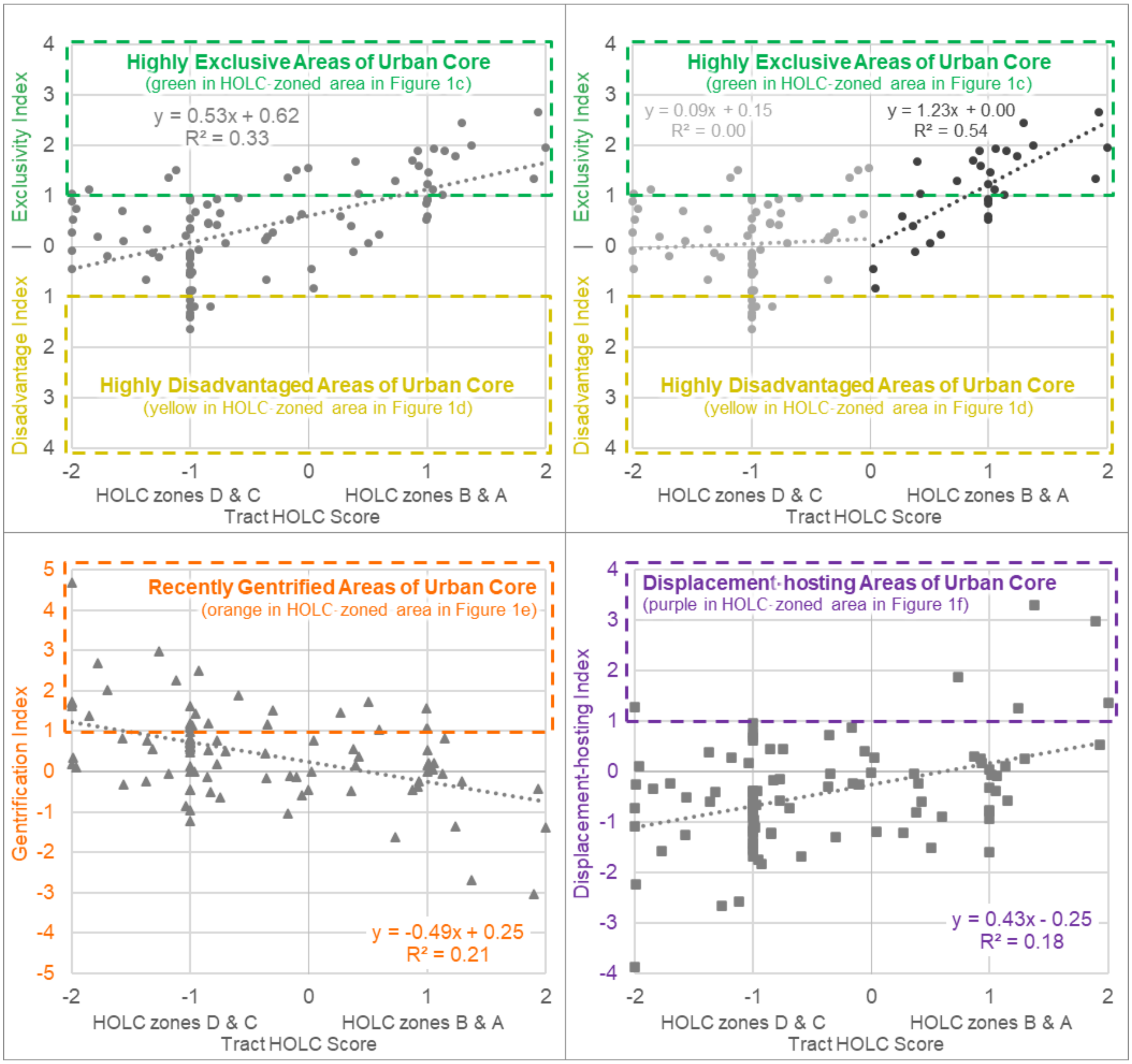

Figure S5. Relations of neighborhood indices to HOLC scores

We cannot emphasize enough that the finding of an apparently positive statistical relationship between historical HOLC privilege (or discrimination) and contemporary persistent exclusivity (or disadvantage) is actually driven entirely by variance in the privileged end of the spectrum (Figure S5, top-right). Rather than being a progressive conclusion regarding the legacies of historical racism, this apparent positive relationship instead actually centers the exclusive, privilege experience and almost entirely neglects the factors responsible for persistent disadvantage, especially as those neighborhoods were 'conveniently' already displaced out of the urban core and so do not show up in any analysis related to historical redlining.

For example, among census tracts predominantly aligned with historical HOLC zones A and B, seven-in-eight (88%) scored above-average (index > 0) on the persistent Exclusivity Index, and six-in-ten (60%) scored as Highly Exclusive (index > 1) areas today (Table S6).

In contrast, tracts predominantly in historical HOLC zones C or D were approximately equally likely to score above-average (index > 0) or below-average (index < 0) on the contemporary Exclusivity Index, and only small portions of these HOLC C and D zones overlapped highly exclusive or highly disadvantaged areas of the last decade (Table S6).

However, only 35% of all the tracts in the study region intersected the small extent of the historical HOLC map covering the urban core. Problematically, at least for making statistical extrapolations from the HOLC area, the subset of urban-core tracts also over-represented the exclusive and gentrified neighborhood typologies and under-represented the disadvantaged and displacement-hosting typologies relative to their frequencies in the overall study region. The great majority of persistently highly disadvantaged and recently strongly displacement-hosting areas are located outside the historically zoned urban core, as are even the majority of persistently highly exclusive areas (Table S7).

We found, therefore, that attempting to predict or characterize contemporary urban neighborhood typologies on the basis of the historical HOLC map was inherently flawed by being both spatially biased, focusing on a too-small and non-representative a subsection of the city, and regressively biased, by accidentally mathematically emphasizing the strength of the relationship between historically privileged zones and contemporarily exclusive areas more than the weakness of the relationship between historically disinvested zones and contemporarily disadvantaged areas.

Rather than the historically disinvested HOLC C and D zones being strongly related to contemporarily disadvantaged areas, they were instead strongly predictive of recent gentrification. For example, more than two-thirds (70%) of the census tracts aligned with former HOLC zones C and D scored above-average in the contemporary Gentrification Index, i.e., gained population of relative socioeconomic advantage from 2010-2020; of these tracts, nearly all (95%) also scored below-average on the Displacement-hosting Index, i.e., lost population of relative socioeconomic disadvantage. This stands in stark contrast to the premise in much recent urban social and environmental justice discussion that historically redlined urban areas do and will exhibit persisting, or even increasing, proportions of historically marginalized demographic groups today.

In this study, fewer than a quarter (23%) of tracts aligned with former HOLC C or D zones showed the increasing proportions of historically marginalized groups characteristic of displacement-hosting areas, from 2010-2020. Rather than communities of socioeconomically disadvantaged residents persisting primarily in formerly redlined areas, we instead find these communities already residing entirely outside the envelope covered by the historical HOLC template (see manuscript Figure 1d). This highlights the importance of the ninety years of development since the height of redlining in this comparatively young city, which had already pushed communities characterized by socioeconomic disadvantage out of the urban core, but in which communities those socioeconomic disadvantages still persist even over the most recent decade. In contrast to these established communities, which are still experiencing persistent socioeconomic disadvantage but reside in coherent clusters and often just outside the historically regulated urban core (Figure 1d), more recent displacement of marginalized demographic groups has been to areas even more scattered and further afield (see manuscript Figure 1f).

Table S6. Contemporary fates of historical HOLC zones

top: Proportion of tracts aligned with HOLC zone A (HOLC Score > 1), B (HOLC Score > 0 and <= 1), C (HOLC Score < 0 and >= -1), or D (HOLC Score < -1) that were categorized as persistently highly exclusive (persistent exclusivity index >1), persistently highly disadvantaged (persistent exclusivity index < -1), recently strongly gentrified (gentrification index > 1), or recently strongly displacement-hosting (displacement-hosting index > 1).

bottom: Same, but with HOLC-privileged (A & B) and HOLC-discriminated (C & D) zones combined.

Rows can sum to > 100% because tracts can score highly on more than one of the indices

Tracts in this zone:		highly exclusive	highly disadvantaged	highly gentrifying	highly displacement-hosting	none of these
A	became ->	100%	0%	0%	36%	0%
B	became ->	37%	0%	32%	5%	32%
C	became ->	6%	17%	26%	0%	55%
D	became ->	20%	0%	45%	5%	45%
A & B	became ->	60%	0%	20%	17%	20%
C & D	became ->	10%	12%	31%	1%	52%

Table S7. Apparent origins of contemporary neighborhood typologies among HOLC zones

Proportion of tracts categorized as persistently highly exclusive (persistent exclusivity index >1), persistently highly disadvantaged (persistent exclusivity index < -1), recently strongly gentrified (gentrification index > 1), or recently strongly displacement-hosting (displacement-hosting index > 1) that originated among the HOLC zones (zone A HOLC Score > 1, B HOLC Score > 0 and <= 1, C HOLC Score < 0 and >= -1, D HOLC Score < -1) or outside the historically-zoned urban core area.

Tracts of this typology (index > 1):		A	B	C	D	outside the urban core
highly exclusive	started as ->	21%	13%	6%	8%	53%
highly disadvantaged	started as ->	0%	0%	13%	0%	87%
highly gentrified	started as ->	0%	13%	26%	20%	41%
highly displacement-hosting	started as ->	9%	2%	0%	2%	86%

F. Spotlight on health insurance rates

Socioeconomically-aligned disparities in health were likely further compounded by differential health insurance coverage, with 3.4-times (9%) more uninsured residents in disadvantaged neighborhoods, on average, than in highly exclusive areas in 2020.

This persistent insurance gap remained in the Portland metro study region in 2020 despite the U.S. Affordable Care Act having greatly reduced the number of uninsured over the preceding decade, from 17% to 7% overall across the tri-county area of the Portland metro. We calculated this change in overall rates of uninsurance by filtering resources at data.census.gov to Health Insurance and to Oregon: Counties: Clackamas, Multnomah, and Washington, resulting in data from Table S2701 for 2010 (28) and for 2020 (29) as shown below (Table S8).

Table S8. Proportion of population without health insurance, 2010 and 2020

2010	total pop.	pop. insured	pop. uninsured	% insured	% uninsured
	3,799,107	3,147,603	651,504	83%	17%
2020	total pop.	pop. insured	pop. uninsured	% insured	% uninsured
	4,135,531	3,861,117	274,414	93%	7%

The census tracts in this study that had been among the many that exhibited a high fraction of uninsured residents in 2013 (>15.4%; Figure S6) included the majority of those that persisted with the highest proportions of uninsured residents in 2020 (>13.3% uninsured; Figure S7). However many of the neighborhoods with a high rate of uninsured in 2013 saw their uninsured rate fall to below 9.5% by 2020, whereas the neighborhoods highlighted in Figure S7 did not.

The difference: the great majority of areas with persistently high uninsured rates in both 2013 and 2020 were geographically coincident with our identified persistently disadvantaged neighborhoods (see manuscript Figure 1d and Figure S8, below), whereas the great majority of areas that saw high uninsurance rates in 2013 reduced to low levels by 2020 were “average” areas of the region, which that fell in none of the tails of our neighborhood typology distributions (i.e., white areas in manuscript Figure 1g). Both highly disadvantaged and strongly displacement-hosting areas with high uninsurance rates in 2013 were much more likely to still persist with high rates in 2020 than to see them fall (Figure S8).

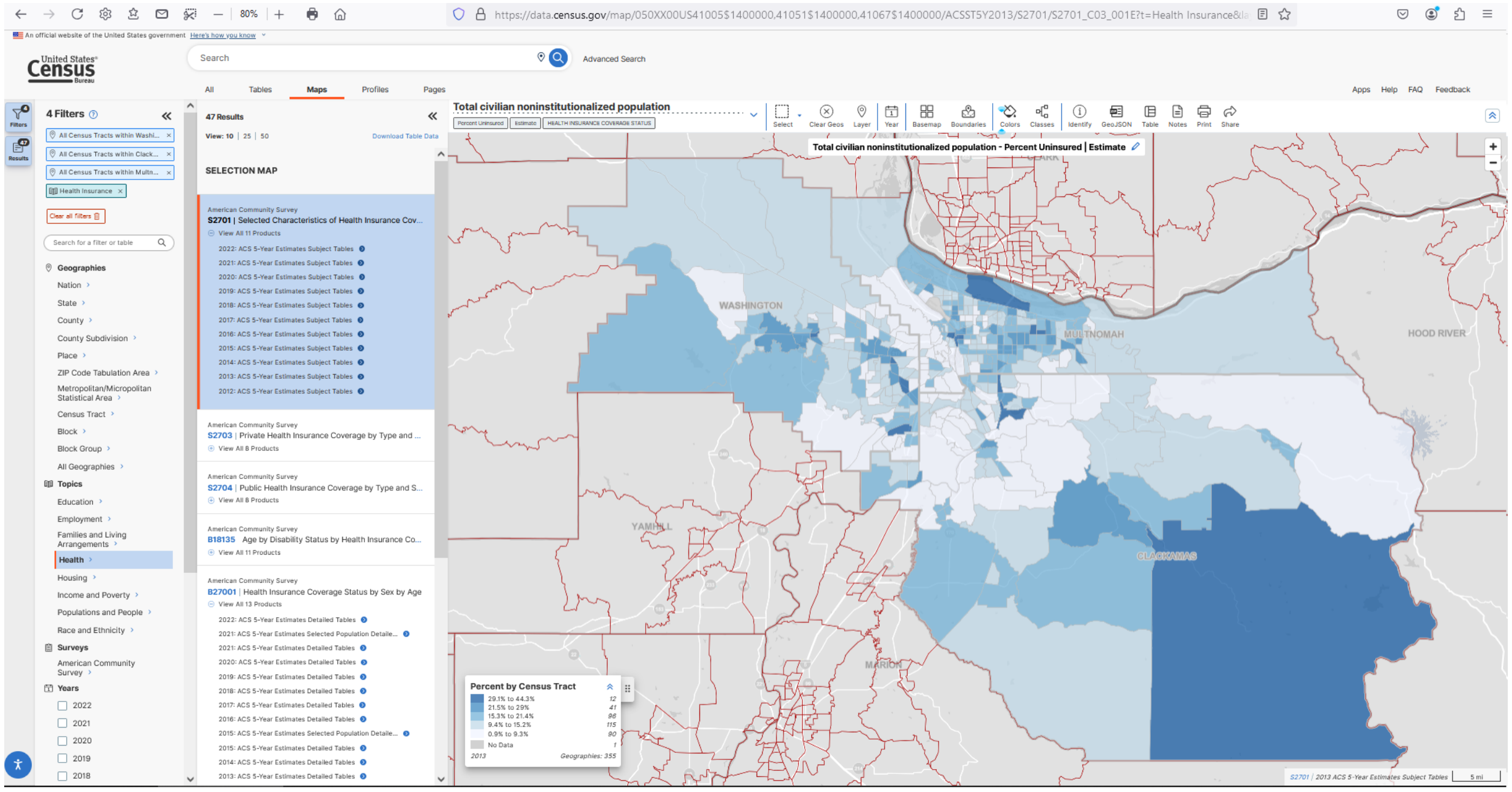

Figure S6. Portland-area Census Tracts map of percent uninsured in 2013.
Screenshot from (30). Map not available for 2010.

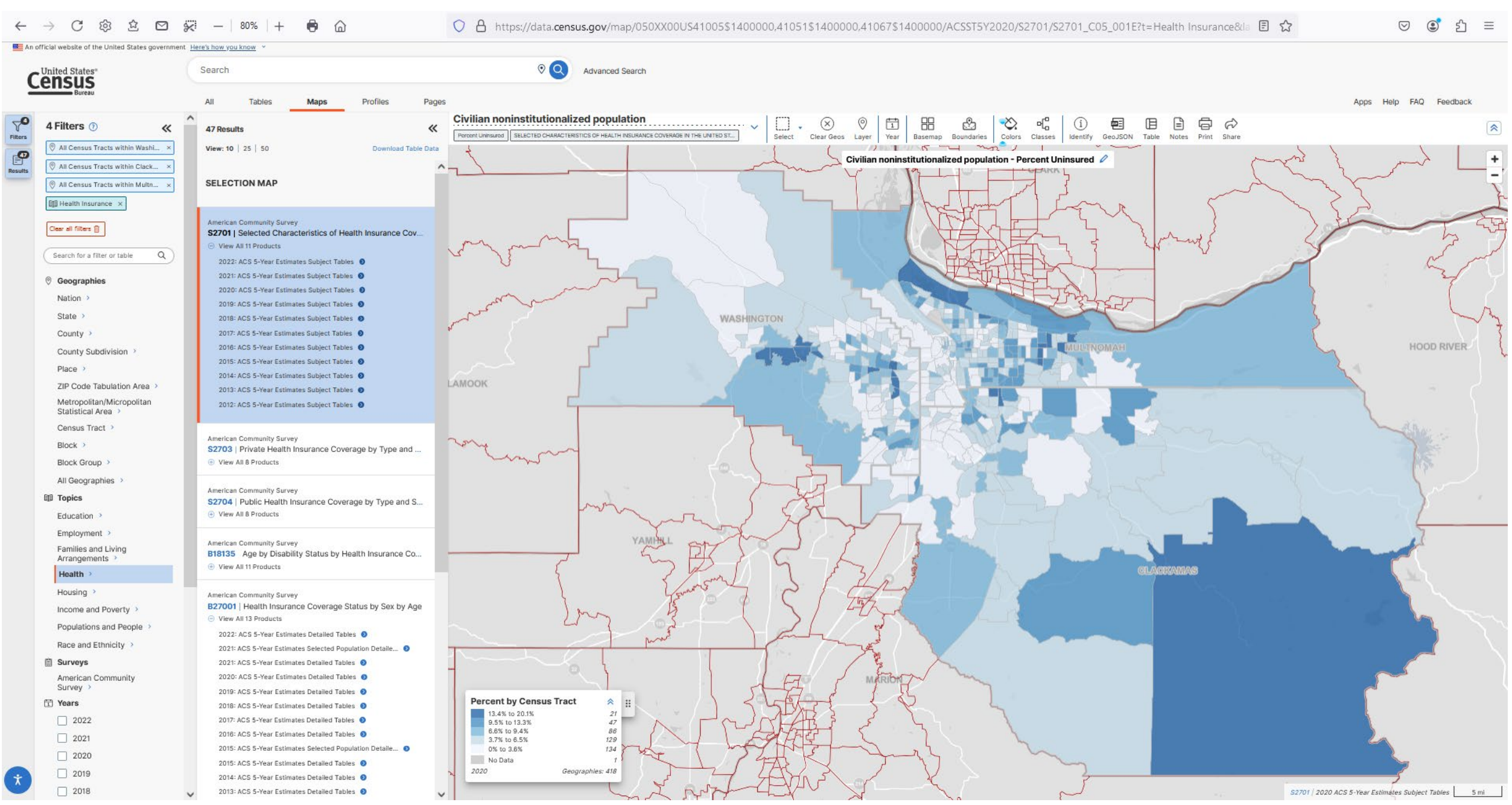

Figure S7. Portland-area Census Tracts map of percent uninsured in 2020.
Screenshot from (31).

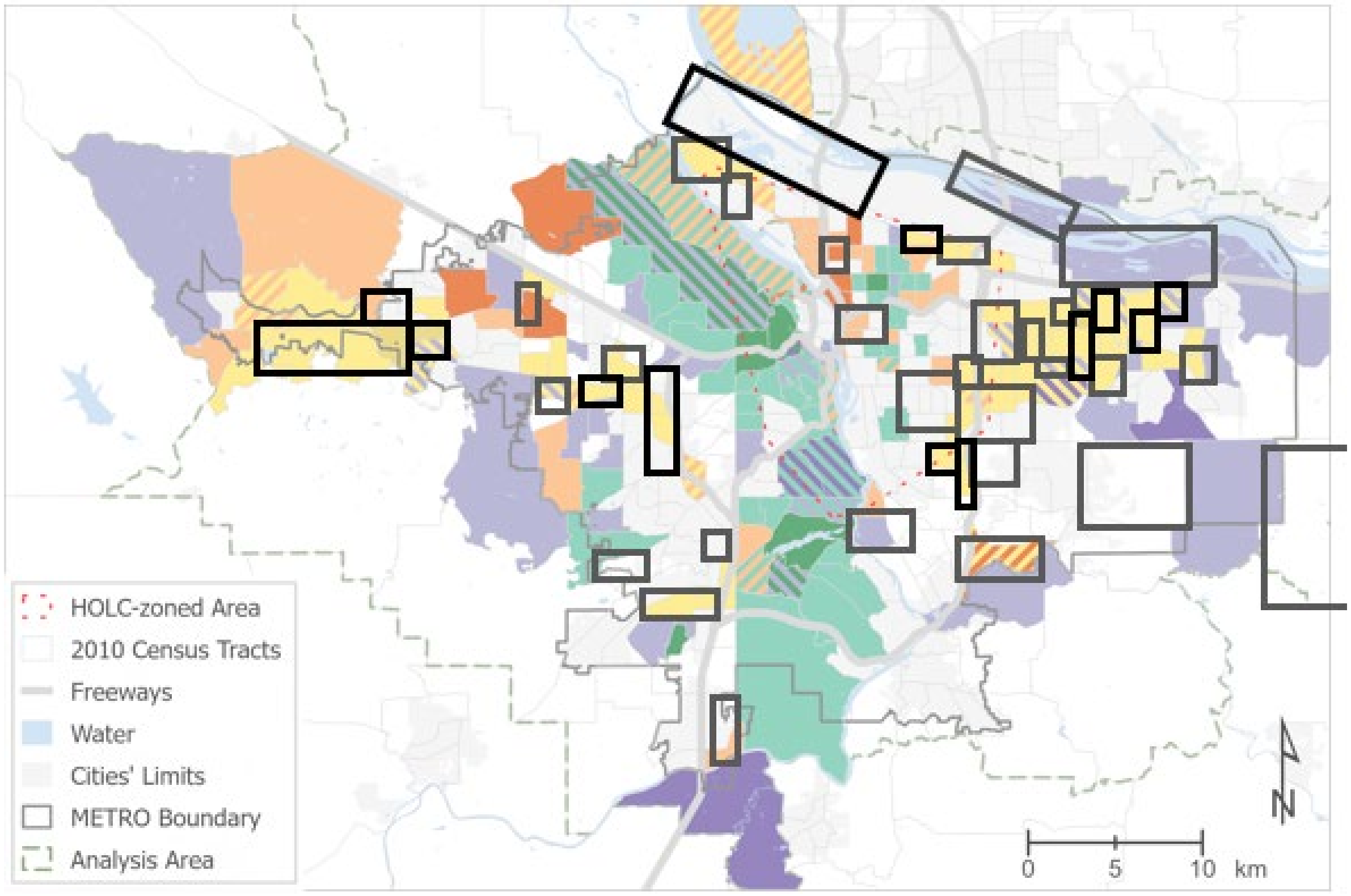

Figure S8. Persistently highly disadvantaged areas also most persistently lack medical insurance between 2013 and 2020.

Figure Legend: Portions of the metro area persisting at 13.4-20.1% uninsurance in 2020 (see Figure S7) are outlined in black; portions persisting at 9.5-12.3% uninsurance in 2020 are outlined in gray boxes. The largest proportion of these areas with persistently high rates of uninsured aligns with persistently disadvantaged areas (yellow). Background census tract colors as in Figure 1g of the manuscript.

REFERENCES

1. US Census Bureau, American Community Survey Data. *Census.gov*. Available at: https://www.census.gov/programs-surveys/acs/data.html [Accessed 11 August 2025].

2. T. Golash-Boza, H. Oh, Crime and Neighborhood Change in the Nation's Capital: From Disinvestment to Gentrification. *Crime & Delinquency* **67**, 1267–1294 (2021).

3. M. S. Mujahid, *et al.*, Gentrification and Displacement in the San Francisco Bay Area: A Comparison of Measurement Approaches. *International Journal of Environmental Research and Public Health* **16**, 2246 (2019).

4. J. R. Sims, Measuring the Effect of Gentrification on Displacement: Multifamily Housing and Eviction in Wisconsin's Madison Urban Region. *Housing Policy Debate* **31**, 736–761 (2021).

5. L. Ding, J. Hwang, E. Divringi, Gentrification and residential mobility in Philadelphia. *Regional Science and Urban Economics* **61**, 38–51 (2016).

6. L. Bates, "Gentrification and Displacement Study: Implementing an Equitable Inclusive Development Strategy in the Context of Gentrification" (Portland State University Library, 2013).

7. T. Thomas, *et al.*, Urban Displacement Replication Project: A Modified Gentrification and Displacement Methodology for the Atlanta, Chicago, Denver, and Memphis SPARCC regions. (2020). Available at: https://www.urbandisplacement.org/wp-content/uploads/2021/08/udp_replication_project_methodology_10.16.2020-converted.pdf.

8. K. Chapple, M. Zuk, Forewarned: The Use of Neighborhood Early Warning Systems for Gentrification and Displacement. *Cityscape* **18**, 109–130 (2016).

9. J. Knight, M. Gharipour, Urban Displacement and Low-Income Communities: The Case of the American City from the Late Twentieth Century. *ArchNet-IJAR* **10**, 06 (2016).

10. M. Zuk, A. H. Bierbaum, K. Chapple, K. Gorska, A. Loukaitou-Sideris, Gentrification, Displacement, and the Role of Public Investment. *Journal of Planning Literature* **33**, 31–44 (2018).

11. K. Chapple, Income Inequality and Urban Displacement: The New Gentrification. *New Labor Forum* **26**, 84–93 (2017).

12. J. Voelkel, D. Hellman, R. Sakuma, V. Shandas, Assessing Vulnerability to Urban Heat: A Study of Disproportionate Heat Exposure and Access to Refuge by Socio-Demographic Status in Portland, Oregon. *International Journal of Environmental Research and Public Health* **15**, 640 (2018).

13. S. L. Cutter, S. Derakhshan, Implementing Disaster Policy: Exploring Scale and Measurement Schemes for Disaster Resilience. *Journal of Homeland Security and Emergency Management* **16** (2019).

14. METRO, Regional Land Information System (RLIS) Data. *Regional Metropolitan (METRO) Planning Organization for the Portland Region*. Available at: https://rlisdiscovery.oregonmetro.gov/ [Accessed 27 February 2024].

15. C. E. Reid, *et al.*, Mapping Community Determinants of Heat Vulnerability. *Environmental Health Perspectives* **117**, 1730–1736 (2009).

16. L. C. Messer, *et al.*, The Development of a Standardized Neighborhood Deprivation Index. *J Urban Health* **83**, 1041–1062 (2006).

17. R. K. Nelson, L. Winling, et al., Mapping Inequality: Redlining in New Deal America. *Digital Scholarship Lab* (2023). Available at: https://dsl.richmond.edu/panorama/redlining/ [Accessed 12 November 2019].

18. USGS, National Land Cover Database: Percent Impervious and Percent Tree Canopy Data, 2011, 2019, 2021. *U.S. Geological Survey*. Available at: https://www.mrlc.gov/data [Accessed 6 August 2024].

19. City of Portland, Tree Inventory Project. Available at: https://www.portland.gov/trees/get-involved/treeinventory [Accessed 9 August 2021].

20. Planet Labs, Planetscope PSOrtho5b Imagery Data. Available at: https://www.planet.com/ [Accessed 1 April 2024].

21. US EPA, EJSCREEN Data Download. *U.S. Environmental Protection Agency*. Available at: https://www.epa.gov/ejscreen/download-ejscreen-data [Accessed 14 June 2024].

22. CAPA, NIHHIS, Heat Watch Portland Metro Region Dataset: rasters_chw_portland_092823.zip. (2023). Available at: https://osf.io/https://osf.io/cx6sk?view_only=cafa95f8fc9d4dd0bf84ed5cad8cd664 [Accessed 23 July 2024].

23. US DOT, National Transportation Noise Map: Continental U.S. road, freight and passenger rail, and aviation noise. *Bureau of Transportation Statistics, U.S. Department of Transportation*. Available at: https://www.bts.gov/geospatial/national-transportation-noise-map [Accessed 15 September 2024].

24. US FEMA, National Risk Index: Data Resources. *U.S. Federal Emergency Management Agency*. Available at: https://hazards.fema.gov/nri/data-resources#csvDownload [Accessed 3 July 2024].

25. US CDC, PLACES: Local Data for Better Health, Census Tract Data, 2023 Release. *US Centers for Disease Control and Prevention*. Available at: https://www.cdc.gov/places/index.html [Accessed 14 June 2024].

26. US Census Bureau, 2018-2022 American Community Survey 5-year Age and Sex Estimates by Tract. *Data downloaded from: Regional Land Information System (RLIS), Regional Metropolitan Planning Organization (METRO) for the Portland Region*. Available at: https://rlisdiscovery.oregonmetro.gov/ [Accessed 23 August 2024].

27. METRO, Urban and rural reserves. *METRO Regional Metropolitan Planning Organization for the Portland Region* (2014). Available at: https://www.oregonmetro.gov/urban-and-rural-reserves [Accessed 12 August 2025].

28. US Census Bureau, ACS Health insurance information by county, Table S2701: 2010 ACS 1-year Estimates Subject Tables: Health Insurance Coverage Status. Available at: https://data.census.gov/table/ACSST1Y2010.S2701?q=health%20insurance%20&g=040XX00US41_050XX00US41005,41051,41067&y=2010 [Accessed 27 October 2024].

29. US Census Bureau, ACS Health insurance information by county, Table S2701: 2020 ACS 5-year Estimates Subject Tables: Selected Characteristics of Health Insurance Coverage in the United States. Available at: https://data.census.gov/table?q=health%20insurance%20&g=040XX00US41_050XX00US41005,41051,41067&y=2020 [Accessed 27 October 2024].

30. US Census Bureau, Selection Map of ACS S2701: Selected Characteristics of Health Insurance Coverage in the United States: Percent by Census Tract (2013). Available at: https://data.census.gov/map/050XX00US41005$1400000,41051$1400000,41067$1400000/ACSST5Y2013/S2701/S2701_C03_001E?t=Health%20Insurance&layer=VT_2013_140_00_PY_D1&loc=45.3347,-122.6218,z9.0543 [Accessed 27 October 2024].

31. US Census Bureau, Selection Map of ACS S2701: Selected Characteristics of Health Insurance Coverage in the United States: Percent by Census Tract (2020). Available at: https://data.census.gov/map/050XX00US41005$1400000,41051$1400000,41067$1400000/ACSST5Y2020/S2701/S2701_C05_001E?t=Health%20Insurance&layer=VT_2020_140_00_PY_D1&loc=45.3347,-122.6216,z9.0541 [Accessed 27 October 2024].
